# Measurements of the $\mathrm {p}\mathrm {p}\rightarrow \mathrm{Z}\mathrm{Z}$ production cross section and the $\mathrm{Z}\rightarrow 4\ell$ branching fraction, and constraints on anomalous triple gauge couplings at $\sqrt{s} = 13\,\text {TeV}$

**DOI:** 10.1140/epjc/s10052-018-5567-9

**Published:** 2018-02-24

**Authors:** A. M. Sirunyan, A. Tumasyan, W. Adam, F Ambrogi, E. Asilar, T. Bergauer, J. Brandstetter, E. Brondolin, M. Dragicevic, J. Erö, M. Flechl, M. Friedl, R. Frühwirth, V. M. Ghete, J. Grossmann, J. Hrubec, M. Jeitler, A. König, N. Krammer, I. Krätschmer, D. Liko, T. Madlener, I. Mikulec, E. Pree, D. Rabady, N. Rad, H. Rohringer, J. Schieck, R. Schöfbeck, M. Spanring, D. Spitzbart, W. Waltenberger, J. Wittmann, C.-E. Wulz, M. Zarucki, V. Chekhovsky, V. Mossolov, J. Suarez Gonzalez, E. A. De Wolf, D. Di Croce, X. Janssen, J. Lauwers, M. Van De Klundert, H. Van Haevermaet, P. Van Mechelen, N. Van Remortel, S. Abu Zeid, F. Blekman, J. D’Hondt, I. De Bruyn, J. De Clercq, K. Deroover, G. Flouris, D. Lontkovskyi, S. Lowette, S. Moortgat, L. Moreels, Q. Python, K. Skovpen, S. Tavernier, W. Van Doninck, P. Van Mulders, I. Van Parijs, H. Brun, B. Clerbaux, G. De Lentdecker, H. Delannoy, G. Fasanella, L. Favart, R. Goldouzian, A. Grebenyuk, G. Karapostoli, T. Lenzi, J. Luetic, T. Maerschalk, A. Marinov, A. Randle-conde, T. Seva, C. Vander Velde, P. Vanlaer, D. Vannerom, R. Yonamine, F. Zenoni, F. Zhang, A. Cimmino, T. Cornelis, D. Dobur, A. Fagot, M. Gul, I. Khvastunov, D. Poyraz, C. Roskas, S. Salva, M. Tytgat, W. Verbeke, N. Zaganidis, H. Bakhshiansohi, O. Bondu, S. Brochet, G. Bruno, C. Caputo, A. Caudron, S. De Visscher, C. Delaere, M. Delcourt, B. Francois, A. Giammanco, A. Jafari, M. Komm, G. Krintiras, V. Lemaitre, A. Magitteri, A. Mertens, M. Musich, K. Piotrzkowski, L. Quertenmont, M. Vidal Marono, S. Wertz, N. Beliy, W. L. Aldá Júnior, F. L. Alves, G. A. Alves, L. Brito, M. Correa Martins Junior, C. Hensel, A. Moraes, M. E. Pol, P. Rebello Teles, E. Belchior Batista Das Chagas, W. Carvalho, J. Chinellato, A. Custódio, E. M. Da Costa, G. G. Da Silveira, D. De Jesus Damiao, S. Fonseca De Souza, L. M. Huertas Guativa, H. Malbouisson, M. Melo De Almeida, C. Mora Herrera, L. Mundim, H. Nogima, A. Santoro, A. Sznajder, E. J. Tonelli Manganote, F. Torres Da Silva De Araujo, A. Vilela Pereira, S. Ahuja, C. A. Bernardes, T. R. Fernandez Perez Tomei, E. M. Gregores, P. G. Mercadante, S. F. Novaes, Sandra S. Padula, D. Romero Abad, J. C. Ruiz Vargas, A. Aleksandrov, R. Hadjiiska, P. Iaydjiev, M. Misheva, M. Rodozov, M. Shopova, S. Stoykova, G. Sultanov, A. Dimitrov, I. Glushkov, L. Litov, B. Pavlov, P. Petkov, W. Fang, X. Gao, M. Ahmad, J. G. Bian, G. M. Chen, H. S. Chen, M. Chen, Y. Chen, C. H. Jiang, D. Leggat, H. Liao, Z. Liu, F. Romeo, S. M. Shaheen, A. Spiezia, J. Tao, C. Wang, Z. Wang, E. Yazgan, H. Zhang, J. Zhao, Y. Ban, G. Chen, Q. Li, S. Liu, Y. Mao, S. J. Qian, D. Wang, Z. Xu, C. Avila, A. Cabrera, L. F. Chaparro Sierra, C. Florez, C. F. González Hernández, J. D. Ruiz Alvarez, B. Courbon, N. Godinovic, D. Lelas, I. Puljak, P. M. Ribeiro Cipriano, T. Sculac, Z. Antunovic, M. Kovac, V. Brigljevic, D. Ferencek, K. Kadija, B. Mesic, A. Starodumov, T. Susa, M. W. Ather, A. Attikis, G. Mavromanolakis, J. Mousa, C. Nicolaou, F. Ptochos, P. A. Razis, H. Rykaczewski, M. Finger, M. Finger, E. Carrera Jarrin, Y. Assran, M. A. Mahmoud, A. Mahrous, R. K. Dewanjee, M. Kadastik, L. Perrini, M. Raidal, A. Tiko, C. Veelken, P. Eerola, J. Pekkanen, M. Voutilainen, J. Härkönen, T. Järvinen, V. Karimäki, R. Kinnunen, T. Lampén, K. Lassila-Perini, S. Lehti, T. Lindén, P. Luukka, E. Tuominen, J. Tuominiemi, E. Tuovinen, J. Talvitie, T. Tuuva, M. Besancon, F. Couderc, M. Dejardin, D. Denegri, J. L. Faure, F. Ferri, S. Ganjour, S. Ghosh, A. Givernaud, P. Gras, G. Hamel de Monchenault, P. Jarry, I. Kucher, E. Locci, M. Machet, J. Malcles, G. Negro, J. Rander, A. Rosowsky, M. Ö. Sahin, M. Titov, A. Abdulsalam, I. Antropov, S. Baffioni, F. Beaudette, P. Busson, L. Cadamuro, C. Charlot, R. Granier de Cassagnac, M. Jo, S. Lisniak, A. Lobanov, J. Martin Blanco, M. Nguyen, C. Ochando, G. Ortona, P. Paganini, P. Pigard, S. Regnard, R. Salerno, J. B. Sauvan, Y. Sirois, A. G. Stahl Leiton, T. Strebler, Y. Yilmaz, A. Zabi, A. Zghiche, J.-L. Agram, J. Andrea, D. Bloch, J.-M. Brom, M. Buttignol, E. C. Chabert, N. Chanon, C. Collard, E. Conte, X. Coubez, J.-C. Fontaine, D. Gelé, U. Goerlach, M. Jansová, A.-C. Le Bihan, N. Tonon, P. Van Hove, S. Gadrat, S. Beauceron, C. Bernet, G. Boudoul, R. Chierici, D. Contardo, P. Depasse, H. El Mamouni, J. Fay, L. Finco, S. Gascon, M. Gouzevitch, G. Grenier, B. Ille, F. Lagarde, I. B. Laktineh, M. Lethuillier, L. Mirabito, A. L. Pequegnot, S. Perries, A. Popov, V. Sordini, M. Vander Donckt, S. Viret, A. Khvedelidze, Z. Tsamalaidze, C. Autermann, S. Beranek, L. Feld, M. K. Kiesel, K. Klein, M. Lipinski, M. Preuten, C. Schomakers, J. Schulz, T. Verlage, A. Albert, E. Dietz-Laursonn, D. Duchardt, M. Endres, M. Erdmann, S. Erdweg, T. Esch, R. Fischer, A. Güth, M. Hamer, T. Hebbeker, C. Heidemann, K. Hoepfner, S. Knutzen, M. Merschmeyer, A. Meyer, P. Millet, S. Mukherjee, M. Olschewski, K. Padeken, T. Pook, M. Radziej, H. Reithler, M. Rieger, F. Scheuch, D. Teyssier, S. Thüer, G. Flügge, B. Kargoll, T. Kress, A. Künsken, J. Lingemann, T. Müller, A. Nehrkorn, A. Nowack, C. Pistone, O. Pooth, A. Stahl, M. Aldaya Martin, T. Arndt, C. Asawatangtrakuldee, K. Beernaert, O. Behnke, U. Behrens, A. Bermúdez Martínez, A. A. Bin Anuar, K. Borras, V. Botta, A. Campbell, P. Connor, C. Contreras-Campana, F. Costanza, C. Diez Pardos, G. Eckerlin, D. Eckstein, T. Eichhorn, E. Eren, E. Gallo, J. Garay Garcia, A. Geiser, A. Gizhko, J. M. Grados Luyando, A. Grohsjean, P. Gunnellini, M. Guthoff, A. Harb, J. Hauk, M. Hempel, H. Jung, A. Kalogeropoulos, M. Kasemann, J. Keaveney, C. Kleinwort, I. Korol, D. Krücker, W. Lange, A. Lelek, T. Lenz, J. Leonard, K. Lipka, W. Lohmann, R. Mankel, I.-A. Melzer-Pellmann, A. B. Meyer, G. Mittag, J. Mnich, A. Mussgiller, E. Ntomari, D. Pitzl, A. Raspereza, B. Roland, M. Savitskyi, P. Saxena, R. Shevchenko, S. Spannagel, N. Stefaniuk, G. P. Van Onsem, R. Walsh, Y. Wen, K. Wichmann, C. Wissing, O. Zenaiev, S. Bein, V. Blobel, M. Centis Vignali, T. Dreyer, E. Garutti, D. Gonzalez, J. Haller, A. Hinzmann, M. Hoffmann, A. Karavdina, R. Klanner, R. Kogler, N. Kovalchuk, S. Kurz, T. Lapsien, I. Marchesini, D. Marconi, M. Meyer, M. Niedziela, D. Nowatschin, F. Pantaleo, T. Peiffer, A. Perieanu, C. Scharf, P. Schleper, A. Schmidt, S. Schumann, J. Schwandt, J. Sonneveld, H. Stadie, G. Steinbrück, F. M. Stober, M. Stöver, H. Tholen, D. Troendle, E. Usai, L. Vanelderen, A. Vanhoefer, B. Vormwald, M. Akbiyik, C. Barth, S. Baur, E. Butz, R. Caspart, T. Chwalek, F. Colombo, W. De Boer, A. Dierlamm, B. Freund, R. Friese, M. Giffels, A. Gilbert, D. Haitz, F. Hartmann, S. M. Heindl, U. Husemann, F. Kassel, S. Kudella, H. Mildner, M. U. Mozer, Th. Müller, M. Plagge, G. Quast, K. Rabbertz, M. Schröder, I. Shvetsov, G. Sieber, H. J. Simonis, R. Ulrich, S. Wayand, M. Weber, T. Weiler, S. Williamson, C. Wöhrmann, R. Wolf, G. Anagnostou, G. Daskalakis, T. Geralis, V. A. Giakoumopoulou, A. Kyriakis, D. Loukas, I. Topsis-Giotis, G. Karathanasis, S. Kesisoglou, A. Panagiotou, N. Saoulidou, K. Kousouris, I. Evangelou, C. Foudas, P. Kokkas, S. Mallios, N. Manthos, I. Papadopoulos, E. Paradas, J. Strologas, F. A. Triantis, M. Csanad, N. Filipovic, G. Pasztor, G. I. Veres, G. Bencze, C. Hajdu, D. Horvath, Á. Hunyadi, F. Sikler, V. Veszpremi, G. Vesztergombi, A. J. Zsigmond, N. Beni, S. Czellar, J. Karancsi, A. Makovec, J. Molnar, Z. Szillasi, M. Bartók, P. Raics, Z. L. Trocsanyi, B. Ujvari, S. Choudhury, J. R. Komaragiri, S. Bahinipati, S. Bhowmik, P. Mal, K. Mandal, A. Nayak, D. K. Sahoo, N. Sahoo, S. K. Swain, S. Bansal, S. B. Beri, V. Bhatnagar, R. Chawla, N. Dhingra, A. K. Kalsi, A. Kaur, M. Kaur, R. Kumar, P. Kumari, A. Mehta, J. B. Singh, G. Walia, Ashok Kumar, Aashaq Shah, A. Bhardwaj, S. Chauhan, B. C. Choudhary, R. B. Garg, S. Keshri, A. Kumar, S. Malhotra, M. Naimuddin, K. Ranjan, R. Sharma, R. Bhardwaj, R. Bhattacharya, S. Bhattacharya, U. Bhawandeep, S. Dey, S. Dutt, S. Dutta, S. Ghosh, N. Majumdar, A. Modak, K. Mondal, S. Mukhopadhyay, S. Nandan, A. Purohit, A. Roy, D. Roy, S. Roy Chowdhury, S. Sarkar, M. Sharan, S. Thakur, P. K. Behera, R. Chudasama, D. Dutta, V. Jha, V. Kumar, A. K. Mohanty, P. K. Netrakanti, L. M. Pant, P. Shukla, A. Topkar, T. Aziz, S. Dugad, B. Mahakud, S. Mitra, G. B. Mohanty, N. Sur, B. Sutar, S. Banerjee, S. Bhattacharya, S. Chatterjee, P. Das, M. Guchait, Sa. Jain, S. Kumar, M. Maity, G. Majumder, K. Mazumdar, T. Sarkar, N. Wickramage, S. Chauhan, S. Dube, V. Hegde, A. Kapoor, K. Kothekar, S. Pandey, A. Rane, S. Sharma, S. Chenarani, E. Eskandari Tadavani, S. M. Etesami, M. Khakzad, M. Mohammadi Najafabadi, M. Naseri, S. Paktinat Mehdiabadi, F. Rezaei Hosseinabadi, B. Safarzadeh, M. Zeinali, M. Felcini, M. Grunewald, M. Abbrescia, C. Calabria, A. Colaleo, D. Creanza, L. Cristella, N. De Filippis, M. De Palma, F. Errico, L. Fiore, G. Iaselli, S. Lezki, G. Maggi, M. Maggi, G. Miniello, S. My, S. Nuzzo, A. Pompili, G. Pugliese, R. Radogna, A. Ranieri, G. Selvaggi, A. Sharma, L. Silvestris, R. Venditti, P. Verwilligen, G. Abbiendi, C. Battilana, D. Bonacorsi, S. Braibant-Giacomelli, R. Campanini, P. Capiluppi, A. Castro, F. R. Cavallo, S. S. Chhibra, G. Codispoti, M. Cuffiani, G. M. Dallavalle, F. Fabbri, A. Fanfani, D. Fasanella, P. Giacomelli, C. Grandi, L. Guiducci, S. Marcellini, G. Masetti, A. Montanari, F. L. Navarria, A. Perrotta, A. M. Rossi, T. Rovelli, G. P. Siroli, N. Tosi, S. Albergo, S. Costa, A. Di Mattia, F. Giordano, R. Potenza, A. Tricomi, C. Tuve, G. Barbagli, K. Chatterjee, V. Ciulli, C. Civinini, R. D’Alessandro, E. Focardi, P. Lenzi, M. Meschini, S. Paoletti, L. Russo, G. Sguazzoni, D. Strom, L. Viliani, L. Benussi, S. Bianco, F. Fabbri, D. Piccolo, F. Primavera, V. Calvelli, F. Ferro, E. Robutti, S. Tosi, A. Benaglia, L. Brianza, F. Brivio, V. Ciriolo, M. E. Dinardo, S. Fiorendi, S. Gennai, A. Ghezzi, P. Govoni, M. Malberti, S. Malvezzi, R. A. Manzoni, D. Menasce, L. Moroni, M. Paganoni, D. Pedrini, S. Pigazzini, S. Ragazzi, T. Tabarelli de Fatis, S. Buontempo, N. Cavallo, S. Di Guida, F. Fabozzi, F. Fienga, A. O. M. Iorio, W. A. Khan, L. Lista, S. Meola, P. Paolucci, C. Sciacca, F. Thyssen, P. Azzi, N. Bacchetta, L. Benato, D. Bisello, A. Boletti, R. Carlin, A. Carvalho Antunes De Oliveira, P. Checchia, M. Dall’Osso, P. De Castro Manzano, T. Dorigo, U. Dosselli, U. Gasparini, A. Gozzelino, S. Lacaprara, P. Lujan, M. Margoni, A. T. Meneguzzo, N. Pozzobon, P. Ronchese, R. Rossin, F. Simonetto, E. Torassa, S. Ventura, M. Zanetti, P. Zotto, A. Braghieri, A. Magnani, P. Montagna, S. P. Ratti, V. Re, M. Ressegotti, C. Riccardi, P. Salvini, I. Vai, P. Vitulo, L. Alunni Solestizi, M. Biasini, G. M. Bilei, C. Cecchi, D. Ciangottini, L. Fanò, P. Lariccia, R. Leonardi, E. Manoni, G. Mantovani, V. Mariani, M. Menichelli, A. Rossi, A. Santocchia, D. Spiga, K. Androsov, P. Azzurri, G. Bagliesi, J. Bernardini, T. Boccali, L. Borrello, R. Castaldi, M. A. Ciocci, R. Dell’Orso, G. Fedi, L. Giannini, A. Giassi, M. T. Grippo, F. Ligabue, T. Lomtadze, E. Manca, G. Mandorli, L. Martini, A. Messineo, F. Palla, A. Rizzi, A. Savoy-Navarro, P. Spagnolo, R. Tenchini, G. Tonelli, A. Venturi, P. G. Verdini, L. Barone, F. Cavallari, M. Cipriani, N. Daci, D. Del Re, E. Di Marco, M. Diemoz, S. Gelli, E. Longo, F. Margaroli, B. Marzocchi, P. Meridiani, G. Organtini, R. Paramatti, F. Preiato, S. Rahatlou, C. Rovelli, F. Santanastasio, N. Amapane, R. Arcidiacono, S. Argiro, M. Arneodo, N. Bartosik, R. Bellan, C. Biino, N. Cartiglia, F. Cenna, M. Costa, R. Covarelli, A. Degano, N. Demaria, B. Kiani, C. Mariotti, S. Maselli, E. Migliore, V. Monaco, E. Monteil, M. Monteno, M. M. Obertino, L. Pacher, N. Pastrone, M. Pelliccioni, G. L. Pinna Angioni, F. Ravera, A. Romero, M. Ruspa, R. Sacchi, K. Shchelina, V. Sola, A. Solano, A. Staiano, P. Traczyk, S. Belforte, M. Casarsa, F. Cossutti, G. Della Ricca, A. Zanetti, D. H. Kim, G. N. Kim, M. S. Kim, J. Lee, S. Lee, S. W. Lee, C. S. Moon, Y. D. Oh, S. Sekmen, D. C. Son, Y. C. Yang, A. Lee, H. Kim, D. H. Moon, G. Oh, J. A. Brochero Cifuentes, J. Goh, T. J. Kim, S. Cho, S. Choi, Y. Go, D. Gyun, S. Ha, B. Hong, Y. Jo, Y. Kim, K. Lee, K. S. Lee, S. Lee, J. Lim, S. K. Park, Y. Roh, J. Almond, J. Kim, J. S. Kim, H. Lee, K. Lee, K. Nam, S. B. Oh, B. C. Radburn-Smith, S. h. Seo, U. K. Yang, H. D. Yoo, G. B. Yu, M. Choi, H. Kim, J. H. Kim, J. S. H. Lee, I. C. Park, Y. Choi, C. Hwang, J. Lee, I. Yu, V. Dudenas, A. Juodagalvis, J. Vaitkus, I. Ahmed, Z. A. Ibrahim, M. A. B. Md Ali, F. Mohamad Idris, W. A. T. Wan Abdullah, M. N. Yusli, Z. Zolkapli, R. Reyes-Almanza, G. Ramirez-Sanchez, M. C. Duran-Osuna, H. Castilla-Valdez, E. De La Cruz-Burelo, I. Heredia-De La Cruz, R. I. Rabadan-Trejo, R. Lopez-Fernandez, J. Mejia Guisao, A. Sanchez-Hernandez, S. Carrillo Moreno, C. Oropeza Barrera, F. Vazquez Valencia, I. Pedraza, H. A. Salazar Ibarguen, C. Uribe Estrada, A. Morelos Pineda, D. Krofcheck, P. H. Butler, A. Ahmad, M. Ahmad, Q. Hassan, H. R. Hoorani, A. Saddique, M. A. Shah, M. Shoaib, M. Waqas, H. Bialkowska, M. Bluj, B. Boimska, T. Frueboes, M. Górski, M. Kazana, K. Nawrocki, M. Szleper, P. Zalewski, K. Bunkowski, A. Byszuk, K. Doroba, A. Kalinowski, M. Konecki, J. Krolikowski, M. Misiura, M. Olszewski, A. Pyskir, M. Walczak, P. Bargassa, C. Beirão Da Cruz E. Silva, A. Di Francesco, P. Faccioli, B. Galinhas, M. Gallinaro, J. Hollar, N. Leonardo, L. Lloret Iglesias, M. V. Nemallapudi, J. Seixas, G. Strong, O. Toldaiev, D. Vadruccio, J. Varela, S. Afanasiev, P. Bunin, M. Gavrilenko, I. Golutvin, I. Gorbunov, A. Kamenev, V. Karjavin, A. Lanev, A. Malakhov, V. Matveev, V. Palichik, V. Perelygin, S. Shmatov, S. Shulha, N. Skatchkov, V. Smirnov, N. Voytishin, A. Zarubin, Y. Ivanov, V. Kim, E. Kuznetsova, P. Levchenko, V. Murzin, V. Oreshkin, I. Smirnov, V. Sulimov, L. Uvarov, S. Vavilov, A. Vorobyev, Yu. Andreev, A. Dermenev, S. Gninenko, N. Golubev, A. Karneyeu, M. Kirsanov, N. Krasnikov, A. Pashenkov, D. Tlisov, A. Toropin, V. Epshteyn, V. Gavrilov, N. Lychkovskaya, V. Popov, I. Pozdnyakov, G. Safronov, A. Spiridonov, A. Stepennov, M. Toms, E. Vlasov, A. Zhokin, T. Aushev, A. Bylinkin, M. Chadeeva, P. Parygin, D. Philippov, S. Polikarpov, E. Popova, V. Rusinov, V. Andreev, M. Azarkin, I. Dremin, M. Kirakosyan, A. Terkulov, A. Baskakov, A. Belyaev, E. Boos, M. Dubinin, L. Dudko, A. Ershov, A. Gribushin, V. Klyukhin, O. Kodolova, I. Lokhtin, I. Miagkov, S. Obraztsov, S. Petrushanko, V. Savrin, A. Snigirev, V. Blinov, Y. Skovpen, D. Shtol, I. Azhgirey, I. Bayshev, S. Bitioukov, D. Elumakhov, V. Kachanov, A. Kalinin, D. Konstantinov, V. Krychkine, V. Petrov, R. Ryutin, A. Sobol, S. Troshin, N. Tyurin, A. Uzunian, A. Volkov, P. Adzic, P. Cirkovic, D. Devetak, M. Dordevic, J. Milosevic, V. Rekovic, J. Alcaraz Maestre, M. Barrio Luna, M. Cerrada, N. Colino, B. De La Cruz, A. Delgado Peris, A. Escalante Del Valle, C. Fernandez Bedoya, J. P. Fernández Ramos, J. Flix, M. C. Fouz, P. Garcia-Abia, O. Gonzalez Lopez, S. Goy Lopez, J. M. Hernandez, M. I. Josa, A. Pérez-Calero Yzquierdo, J. Puerta Pelayo, A. Quintario Olmeda, I. Redondo, L. Romero, M. S. Soares, A. Álvarez Fernández, C. Albajar, J. F. de Trocóniz, M. Missiroli, D. Moran, J. Cuevas, C. Erice, J. Fernandez Menendez, I. Gonzalez Caballero, J. R. González Fernández, E. Palencia Cortezon, S. Sanchez Cruz, I. Suárez Andrés, P. Vischia, J. M. Vizan Garcia, I. J. Cabrillo, A. Calderon, B. Chazin Quero, E. Curras, J. Duarte Campderros, M. Fernandez, J. Garcia-Ferrero, G. Gomez, A. Lopez Virto, J. Marco, C. Martinez Rivero, P. Martinez Ruiz del Arbol, F. Matorras, J. Piedra Gomez, T. Rodrigo, A. Ruiz-Jimeno, L. Scodellaro, N. Trevisani, I. Vila, R. Vilar Cortabitarte, D. Abbaneo, E. Auffray, P. Baillon, A. H. Ball, D. Barney, M. Bianco, P. Bloch, A. Bocci, C. Botta, T. Camporesi, R. Castello, M. Cepeda, G. Cerminara, E. Chapon, Y. Chen, D. d’Enterria, A. Dabrowski, V. Daponte, A. David, M. De Gruttola, A. De Roeck, M. Dobson, B. Dorney, T. du Pree, M. Dünser, N. Dupont, A. Elliott-Peisert, P. Everaerts, F. Fallavollita, G. Franzoni, J. Fulcher, W. Funk, D. Gigi, K. Gill, F. Glege, D. Gulhan, P. Harris, J. Hegeman, V. Innocente, P. Janot, O. Karacheban, J. Kieseler, H. Kirschenmann, V. Knünz, A. Kornmayer, M. J. Kortelainen, M. Krammer, C. Lange, P. Lecoq, C. Lourenço, M. T. Lucchini, L. Malgeri, M. Mannelli, A. Martelli, F. Meijers, J. A. Merlin, S. Mersi, E. Meschi, P. Milenovic, F. Moortgat, M. Mulders, H. Neugebauer, S. Orfanelli, L. Orsini, L. Pape, E. Perez, M. Peruzzi, A. Petrilli, G. Petrucciani, A. Pfeiffer, M. Pierini, A. Racz, T. Reis, F. Riva, G. Rolandi, M. Rovere, H. Sakulin, C. Schäfer, C. Schwick, M. Seidel, M. Selvaggi, A. Sharma, P. Silva, P. Sphicas, A. Stakia, J. Steggemann, M. Stoye, M. Tosi, D. Treille, A. Triossi, A. Tsirou, V. Veckalns, M. Verweij, W. D. Zeuner, W. Bertl, L. Caminada, K. Deiters, W. Erdmann, R. Horisberger, Q. Ingram, H. C. Kaestli, D. Kotlinski, U. Langenegger, T. Rohe, S. A. Wiederkehr, F. Bachmair, L. Bäni, P. Berger, L. Bianchini, B. Casal, G. Dissertori, M. Dittmar, M. Donegà, C. Grab, C. Heidegger, D. Hits, J. Hoss, G. Kasieczka, T. Klijnsma, W. Lustermann, B. Mangano, M. Marionneau, M. T. Meinhard, D. Meister, F. Micheli, P. Musella, F. Nessi-Tedaldi, F. Pandolfi, J. Pata, F. Pauss, G. Perrin, L. Perrozzi, M. Quittnat, M. Reichmann, M. Schönenberger, L. Shchutska, V. R. Tavolaro, K. Theofilatos, M. L. Vesterbacka Olsson, R. Wallny, D. H. Zhu, T. K. Aarrestad, C. Amsler, M. F. Canelli, A. De Cosa, R. Del Burgo, S. Donato, C. Galloni, T. Hreus, B. Kilminster, J. Ngadiuba, D. Pinna, G. Rauco, P. Robmann, D. Salerno, C. Seitz, Y. Takahashi, A. Zucchetta, V. Candelise, T. H. Doan, Sh. Jain, R. Khurana, C. M. Kuo, W. Lin, A. Pozdnyakov, S. S. Yu, Arun Kumar, P. Chang, Y. Chao, K. F. Chen, P. H. Chen, F. Fiori, W.-S. Hou, Y. Hsiung, Y. F. Liu, R.-S. Lu, E. Paganis, A. Psallidas, A. Steen, J. f. Tsai, B. Asavapibhop, K. Kovitanggoon, G. Singh, N. Srimanobhas, F. Boran, S. Cerci, S. Damarseckin, Z. S. Demiroglu, C. Dozen, I. Dumanoglu, S. Girgis, G. Gokbulut, Y. Guler, I. Hos, E. E. Kangal, O. Kara, A. Kayis Topaksu, U. Kiminsu, M. Oglakci, G. Onengut, K. Ozdemir, D. Sunar Cerci, B. Tali, S. Turkcapar, I. S. Zorbakir, C. Zorbilmez, B. Bilin, G. Karapinar, K. Ocalan, M. Yalvac, M. Zeyrek, E. Gülmez, M. Kaya, O. Kaya, S. Tekten, E. A. Yetkin, M. N. Agaras, S. Atay, A. Cakir, K. Cankocak, B. Grynyov, L. Levchuk, P. Sorokin, R. Aggleton, F. Ball, L. Beck, J. J. Brooke, D. Burns, E. Clement, D. Cussans, O. Davignon, H. Flacher, J. Goldstein, M. Grimes, G. P. Heath, H. F. Heath, J. Jacob, L. Kreczko, C. Lucas, D. M. Newbold, S. Paramesvaran, A. Poll, T. Sakuma, S. Seif El Nasr-storey, D. Smith, V. J. Smith, K. W. Bell, A. Belyaev, C. Brew, R. M. Brown, L. Calligaris, D. Cieri, D. J. A. Cockerill, J. A. Coughlan, K. Harder, S. Harper, E. Olaiya, D. Petyt, C. H. Shepherd-Themistocleous, A. Thea, I. R. Tomalin, T. Williams, G. Auzinger, R. Bainbridge, S. Breeze, O. Buchmuller, A. Bundock, S. Casasso, M. Citron, D. Colling, L. Corpe, P. Dauncey, G. Davies, A. De Wit, M. Della Negra, R. Di Maria, A. Elwood, Y. Haddad, G. Hall, G. Iles, T. James, R. Lane, C. Laner, L. Lyons, A.-M. Magnan, S. Malik, L. Mastrolorenzo, T. Matsushita, J. Nash, A. Nikitenko, V. Palladino, M. Pesaresi, D. M. Raymond, A. Richards, A. Rose, E. Scott, C. Seez, A. Shtipliyski, S. Summers, A. Tapper, K. Uchida, M. Vazquez Acosta, T. Virdee, N. Wardle, D. Winterbottom, J. Wright, S. C. Zenz, J. E. Cole, P. R. Hobson, A. Khan, P. Kyberd, I. D. Reid, P. Symonds, L. Teodorescu, M. Turner, A. Borzou, K. Call, J. Dittmann, K. Hatakeyama, H. Liu, N. Pastika, C. Smith, R. Bartek, A. Dominguez, A. Buccilli, S. I. Cooper, C. Henderson, P. Rumerio, C. West, D. Arcaro, A. Avetisyan, T. Bose, D. Gastler, D. Rankin, C. Richardson, J. Rohlf, L. Sulak, D. Zou, G. Benelli, D. Cutts, A. Garabedian, J. Hakala, U. Heintz, J. M. Hogan, K. H. M. Kwok, E. Laird, G. Landsberg, Z. Mao, M. Narain, J. Pazzini, S. Piperov, S. Sagir, R. Syarif, D. Yu, R. Band, C. Brainerd, D. Burns, M. Calderon De La Barca Sanchez, M. Chertok, J. Conway, R. Conway, P. T. Cox, R. Erbacher, C. Flores, G. Funk, M. Gardner, W. Ko, R. Lander, C. Mclean, M. Mulhearn, D. Pellett, J. Pilot, S. Shalhout, M. Shi, J. Smith, M. Squires, D. Stolp, K. Tos, M. Tripathi, Z. Wang, M. Bachtis, C. Bravo, R. Cousins, A. Dasgupta, A. Florent, J. Hauser, M. Ignatenko, N. Mccoll, D. Saltzberg, C. Schnaible, V. Valuev, E. Bouvier, K. Burt, R. Clare, J. Ellison, J. W. Gary, S. M. A. Ghiasi Shirazi, G. Hanson, J. Heilman, P. Jandir, E. Kennedy, F. Lacroix, O. R. Long, M. Olmedo Negrete, M. I. Paneva, A. Shrinivas, W. Si, L. Wang, H. Wei, S. Wimpenny, B. R. Yates, J. G. Branson, S. Cittolin, M. Derdzinski, R. Gerosa, B. Hashemi, A. Holzner, D. Klein, G. Kole, V. Krutelyov, J. Letts, I. Macneill, M. Masciovecchio, D. Olivito, S. Padhi, M. Pieri, M. Sani, V. Sharma, S. Simon, M. Tadel, A. Vartak, S. Wasserbaech, J. Wood, F. Würthwein, A. Yagil, G. Zevi Della Porta, N. Amin, R. Bhandari, J. Bradmiller-Feld, C. Campagnari, A. Dishaw, V. Dutta, M. Franco Sevilla, C. George, F. Golf, L. Gouskos, J. Gran, R. Heller, J. Incandela, S. D. Mullin, A. Ovcharova, H. Qu, J. Richman, D. Stuart, I. Suarez, J. Yoo, D. Anderson, J. Bendavid, A. Bornheim, J. M. Lawhorn, H. B. Newman, T. Nguyen, C. Pena, M. Spiropulu, J. R. Vlimant, S. Xie, Z. Zhang, R. Y. Zhu, M. B. Andrews, T. Ferguson, T. Mudholkar, M. Paulini, J. Russ, M. Sun, H. Vogel, I. Vorobiev, M. Weinberg, J. P. Cumalat, W. T. Ford, F. Jensen, A. Johnson, M. Krohn, S. Leontsinis, T. Mulholland, K. Stenson, S. R. Wagner, J. Alexander, J. Chaves, J. Chu, S. Dittmer, K. Mcdermott, N. Mirman, J. R. Patterson, A. Rinkevicius, A. Ryd, L. Skinnari, L. Soffi, S. M. Tan, Z. Tao, J. Thom, J. Tucker, P. Wittich, M. Zientek, S. Abdullin, M. Albrow, G. Apollinari, A. Apresyan, A. Apyan, S. Banerjee, L. A. T. Bauerdick, A. Beretvas, J. Berryhill, P. C. Bhat, G. Bolla, K. Burkett, J. N. Butler, A. Canepa, G. B. Cerati, H. W. K. Cheung, F. Chlebana, M. Cremonesi, J. Duarte, V. D. Elvira, J. Freeman, Z. Gecse, E. Gottschalk, L. Gray, D. Green, S. Grünendahl, O. Gutsche, R. M. Harris, S. Hasegawa, J. Hirschauer, Z. Hu, B. Jayatilaka, S. Jindariani, M. Johnson, U. Joshi, B. Klima, B. Kreis, S. Lammel, D. Lincoln, R. Lipton, M. Liu, T. Liu, R. Lopes De Sá, J. Lykken, K. Maeshima, N. Magini, J. M. Marraffino, S. Maruyama, D. Mason, P. McBride, P. Merkel, S. Mrenna, S. Nahn, V. O’Dell, K. Pedro, O. Prokofyev, G. Rakness, L. Ristori, B. Schneider, E. Sexton-Kennedy, A. Soha, W. J. Spalding, L. Spiegel, S. Stoynev, J. Strait, N. Strobbe, L. Taylor, S. Tkaczyk, N. V. Tran, L. Uplegger, E. W. Vaandering, C. Vernieri, M. Verzocchi, R. Vidal, M. Wang, H. A. Weber, A. Whitbeck, D. Acosta, P. Avery, P. Bortignon, D. Bourilkov, A. Brinkerhoff, A. Carnes, M. Carver, D. Curry, R. D. Field, I. K. Furic, J. Konigsberg, A. Korytov, K. Kotov, P. Ma, K. Matchev, H. Mei, G. Mitselmakher, D. Rank, D. Sperka, N. Terentyev, L. Thomas, J. Wang, S. Wang, J. Yelton, Y. R. Joshi, S. Linn, P. Markowitz, J. L. Rodriguez, A. Ackert, T. Adams, A. Askew, S. Hagopian, V. Hagopian, K. F. Johnson, T. Kolberg, G. Martinez, T. Perry, H. Prosper, A. Saha, A. Santra, V. Sharma, R. Yohay, M. M. Baarmand, V. Bhopatkar, S. Colafranceschi, M. Hohlmann, D. Noonan, T. Roy, F. Yumiceva, M. R. Adams, L. Apanasevich, D. Berry, R. R. Betts, R. Cavanaugh, X. Chen, O. Evdokimov, C. E. Gerber, D. A. Hangal, D. J. Hofman, K. Jung, J. Kamin, I. D. Sandoval Gonzalez, M. B. Tonjes, H. Trauger, N. Varelas, H. Wang, Z. Wu, J. Zhang, B. Bilki, W. Clarida, K. Dilsiz, S. Durgut, R. P. Gandrajula, M. Haytmyradov, V. Khristenko, J.-P. Merlo, H. Mermerkaya, A. Mestvirishvili, A. Moeller, J. Nachtman, H. Ogul, Y. Onel, F. Ozok, A. Penzo, C. Snyder, E. Tiras, J. Wetzel, K. Yi, B. Blumenfeld, A. Cocoros, N. Eminizer, D. Fehling, L. Feng, A. V. Gritsan, P. Maksimovic, J. Roskes, U. Sarica, M. Swartz, M. Xiao, C. You, A. Al-bataineh, P. Baringer, A. Bean, S. Boren, J. Bowen, J. Castle, S. Khalil, A. Kropivnitskaya, D. Majumder, W. Mcbrayer, M. Murray, C. Royon, S. Sanders, E. Schmitz, R. Stringer, J. D. Tapia Takaki, Q. Wang, A. Ivanov, K. Kaadze, Y. Maravin, A. Mohammadi, L. K. Saini, N. Skhirtladze, S. Toda, F. Rebassoo, D. Wright, C. Anelli, A. Baden, O. Baron, A. Belloni, B. Calvert, S. C. Eno, C. Ferraioli, N. J. Hadley, S. Jabeen, G. Y. Jeng, R. G. Kellogg, J. Kunkle, A. C. Mignerey, F. Ricci-Tam, Y. H. Shin, A. Skuja, S. C. Tonwar, D. Abercrombie, B. Allen, V. Azzolini, R. Barbieri, A. Baty, R. Bi, S. Brandt, W. Busza, I. A. Cali, M. D’Alfonso, Z. Demiragli, G. Gomez Ceballos, M. Goncharov, D. Hsu, Y. Iiyama, G. M. Innocenti, M. Klute, D. Kovalskyi, Y. S. Lai, Y.-J. Lee, A. Levin, P. D. Luckey, B. Maier, A. C. Marini, C. Mcginn, C. Mironov, S. Narayanan, X. Niu, C. Paus, C. Roland, G. Roland, J. Salfeld-Nebgen, G. S. F. Stephans, K. Tatar, D. Velicanu, J. Wang, T. W. Wang, B. Wyslouch, A. C. Benvenuti, R. M. Chatterjee, A. Evans, P. Hansen, S. Kalafut, Y. Kubota, Z. Lesko, J. Mans, S. Nourbakhsh, N. Ruckstuhl, R. Rusack, J. Turkewitz, J. G. Acosta, S. Oliveros, E. Avdeeva, K. Bloom, D. R. Claes, C. Fangmeier, R. Gonzalez Suarez, R. Kamalieddin, I. Kravchenko, J. Monroy, J. E. Siado, G. R. Snow, B. Stieger, M. Alyari, J. Dolen, A. Godshalk, C. Harrington, I. Iashvili, D. Nguyen, A. Parker, S. Rappoccio, B. Roozbahani, G. Alverson, E. Barberis, A. Hortiangtham, A. Massironi, D. M. Morse, D. Nash, T. Orimoto, R. Teixeira De Lima, D. Trocino, D. Wood, S. Bhattacharya, O. Charaf, K. A. Hahn, N. Mucia, N. Odell, B. Pollack, M. H. Schmitt, K. Sung, M. Trovato, M. Velasco, N. Dev, M. Hildreth, K. Hurtado Anampa, C. Jessop, D. J. Karmgard, N. Kellams, K. Lannon, N. Loukas, N. Marinelli, F. Meng, C. Mueller, Y. Musienko, M. Planer, A. Reinsvold, R. Ruchti, G. Smith, S. Taroni, M. Wayne, M. Wolf, A. Woodard, J. Alimena, L. Antonelli, B. Bylsma, L. S. Durkin, S. Flowers, B. Francis, A. Hart, C. Hill, W. Ji, B. Liu, W. Luo, D. Puigh, B. L. Winer, H. W. Wulsin, S. Cooperstein, O. Driga, P. Elmer, J. Hardenbrook, P. Hebda, S. Higginbotham, D. Lange, J. Luo, D. Marlow, K. Mei, I. Ojalvo, J. Olsen, C. Palmer, P. Piroué, D. Stickland, C. Tully, S. Malik, S. Norberg, A. Barker, V. E. Barnes, S. Das, S. Folgueras, L. Gutay, M. K. Jha, M. Jones, A. W. Jung, A. Khatiwada, D. H. Miller, N. Neumeister, C. C. Peng, J. F. Schulte, J. Sun, F. Wang, W. Xie, T. Cheng, N. Parashar, J. Stupak, A. Adair, B. Akgun, Z. Chen, K. M. Ecklund, F. J. M. Geurts, M. Guilbaud, W. Li, B. Michlin, M. Northup, B. P. Padley, J. Roberts, J. Rorie, Z. Tu, J. Zabel, A. Bodek, P. de Barbaro, R. Demina, Y. t. Duh, T. Ferbel, M. Galanti, A. Garcia-Bellido, J. Han, O. Hindrichs, A. Khukhunaishvili, K. H. Lo, P. Tan, M. Verzetti, R. Ciesielski, K. Goulianos, C. Mesropian, A. Agapitos, J. P. Chou, Y. Gershtein, T. A. Gómez Espinosa, E. Halkiadakis, M. Heindl, E. Hughes, S. Kaplan, R. Kunnawalkam Elayavalli, S. Kyriacou, A. Lath, R. Montalvo, K. Nash, M. Osherson, H. Saka, S. Salur, S. Schnetzer, D. Sheffield, S. Somalwar, R. Stone, S. Thomas, P. Thomassen, M. Walker, A. G. Delannoy, M. Foerster, J. Heideman, G. Riley, K. Rose, S. Spanier, K. Thapa, O. Bouhali, A. Castaneda Hernandez, A. Celik, M. Dalchenko, M. De Mattia, A. Delgado, S. Dildick, R. Eusebi, J. Gilmore, T. Huang, T. Kamon, R. Mueller, Y. Pakhotin, R. Patel, A. Perloff, L. Perniè, D. Rathjens, A. Safonov, A. Tatarinov, K. A. Ulmer, N. Akchurin, J. Damgov, F. De Guio, P. R. Dudero, J. Faulkner, E. Gurpinar, S. Kunori, K. Lamichhane, S. W. Lee, T. Libeiro, T. Peltola, S. Undleeb, I. Volobouev, Z. Wang, S. Greene, A. Gurrola, R. Janjam, W. Johns, C. Maguire, A. Melo, H. Ni, P. Sheldon, S. Tuo, J. Velkovska, Q. Xu, M. W. Arenton, P. Barria, B. Cox, R. Hirosky, A. Ledovskoy, H. Li, C. Neu, T. Sinthuprasith, Y. Wang, E. Wolfe, F. Xia, R. Harr, P. E. Karchin, J. Sturdy, S. Zaleski, M. Brodski, J. Buchanan, C. Caillol, S. Dasu, L. Dodd, S. Duric, B. Gomber, M. Grothe, M. Herndon, A. Hervé, U. Hussain, P. Klabbers, A. Lanaro, A. Levine, K. Long, R. Loveless, G. A. Pierro, G. Polese, T. Ruggles, A. Savin, N. Smith, W. H. Smith, D. Taylor, N. Woods, CMS Collaboration

**Affiliations:** 1 Yerevan Physics Institute, Yerevan, Armenia; 2 Institut für Hochenergiephysik, Vienna, Austria; 3 Institute for Nuclear Problems, Minsk, Belarus; 4 Universiteit Antwerpen, Antwerpen, Belgium; 5 Vrije Universiteit Brussel, Brussel, Belgium; 6 Université Libre de Bruxelles, Bruxelles, Belgium; 7 Ghent University, Ghent, Belgium; 8 Université Catholique de Louvain, Louvain-la-Neuve, Belgium; 9 Université de Mons, Mons, Belgium; 10 Centro Brasileiro de Pesquisas Fisicas, Rio de Janeiro, Brazil; 11 Universidade do Estado do Rio de Janeiro, Rio de Janeiro, Brazil; 12 Universidade Estadual Paulista , Universidade Federal do ABC, São Paulo, Brazil; 13 Institute for Nuclear Research and Nuclear Energy, Bulgaria Academy of Sciences, Sofia, Bulgaria; 14 University of Sofia, Sofia, Bulgaria; 15 Beihang University, Beijing, China; 16 Institute of High Energy Physics, Beijing, China; 17 State Key Laboratory of Nuclear Physics and Technology, Peking University, Beijing, China; 18 Universidad de Los Andes, Bogota, Colombia; 19 University of Split, Faculty of Electrical Engineering, Mechanical Engineering and Naval Architecture, Split, Croatia; 20 University of Split, Faculty of Science, Split, Croatia; 21 Institute Rudjer Boskovic, Zagreb, Croatia; 22 University of Cyprus, Nicosia, Cyprus; 23 Charles University, Prague, Czech Republic; 24 Universidad San Francisco de Quito, Quito, Ecuador; 25 Academy of Scientific Research and Technology of the Arab Republic of Egypt, Egyptian Network of High Energy Physics, Cairo, Egypt; 26 National Institute of Chemical Physics and Biophysics, Tallinn, Estonia; 27 Department of Physics, University of Helsinki, Helsinki, Finland; 28 Helsinki Institute of Physics, Helsinki, Finland; 29 Lappeenranta University of Technology, Lappeenranta, Finland; 30 IRFU, CEA, Université Paris-Saclay, Gif-sur-Yvette, France; 31 Laboratoire Leprince-Ringuet, Ecole Polytechnique, CNRS/IN2P3, Université Paris-Saclay, Palaiseau, France; 32 Université de Strasbourg, CNRS, IPHC UMR 7178, F-67000, Strasbourg, France; 33 Centre de Calcul de l’Institut National de Physique Nucleaire et de Physique des Particules, CNRS/IN2P3, Villeurbanne, France; 34 Université de Lyon, Université Claude Bernard Lyon 1, CNRS-IN2P3, Institut de Physique Nucléaire de Lyon, Villeurbanne, France; 35 Georgian Technical University, Tbilisi, Georgia; 36 Tbilisi State University, Tbilisi, Georgia; 37 RWTH Aachen University, I. Physikalisches Institut, Aachen, Germany; 38 RWTH Aachen University, III. Physikalisches Institut A, Aachen, Germany; 39 RWTH Aachen University, III. Physikalisches Institut B, Aachen, Germany; 40 Deutsches Elektronen-Synchrotron, Hamburg, Germany; 41 University of Hamburg, Hamburg, Germany; 42 Institut für Experimentelle Kernphysik, Karlsruhe, Germany; 43 Institute of Nuclear and Particle Physics (INPP), NCSR Demokritos, Aghia Paraskevi, Greece; 44 National and Kapodistrian University of Athens, Athens, Greece; 45 National Technical University of Athens, Athens, Greece; 46 University of Ioánnina, Ioannina, Greece; 47 MTA-ELTE Lendület CMS Particle and Nuclear Physics Group, Eötvös Loránd University, Budapest, Hungary; 48 Wigner Research Centre for Physics, Budapest, Hungary; 49 Institute of Nuclear Research ATOMKI, Debrecen, Hungary; 50 Institute of Physics, University of Debrecen, Debrecen, Hungary; 51 Indian Institute of Science (IISc), Bangalore, India; 52 National Institute of Science Education and Research, Bhubaneswar, India; 53 Panjab University, Chandigarh, India; 54 University of Delhi, Delhi, India; 55 Saha Institute of Nuclear Physics, HBNI, Kolkata, India; 56 Indian Institute of Technology Madras, Madras, India; 57 Bhabha Atomic Research Centre, Mumbai, India; 58 Tata Institute of Fundamental Research-A, Mumbai, India; 59 Tata Institute of Fundamental Research-B, Mumbai, India; 60 Indian Institute of Science Education and Research (IISER), Pune, India; 61 Institute for Research in Fundamental Sciences (IPM), Tehran, Iran; 62 University College Dublin, Dublin, Ireland; 63 INFN Sezione di Bari , Università di Bari , Politecnico di Bari, Bari, Italy; 64 INFN Sezione di Bologna , Università di Bologna, Bologna, Italy; 65 INFN Sezione di Catania , Università di Catania, Catania, Italy; 66 INFN Sezione di Firenze , Università di Firenze, Firenze, Italy; 67 INFN Laboratori Nazionali di Frascati, Frascati, Italy; 68 INFN Sezione di Genova , Università di Genova, Genova, Italy; 69 INFN Sezione di Milano-Bicocca , Università di Milano-Bicocca, Milano, Italy; 70 INFN Sezione di Napoli , Università di Napoli ’Federico II’ , Napoli, Italy, Università della Basilicata , Potenza, Italy, Università G. Marconi, Roma, Italy; 71 INFN Sezione di Padova , Università di Padova , Padova, Italy, Università di Trento, Trento, Italy; 72 INFN Sezione di Pavia , Università di Pavia, Pavia, Italy; 73 INFN Sezione di Perugia , Università di Perugia, Perugia, Italy; 74 INFN Sezione di Pisa , Università di Pisa , Scuola Normale Superiore di Pisa, Pisa, Italy; 75 INFN Sezione di Roma , Università di Roma, Roma, Italy; 76 INFN Sezione di Torino , Università di Torino , Turin, Italy, Università del Piemonte Orientale, Novara, Italy; 77 INFN Sezione di Trieste , Università di Trieste, Trieste, Italy; 78 Kyungpook National University, Daegu, Korea; 79 Chonbuk National University, Jeonju, Korea; 80 Chonnam National University, Institute for Universe and Elementary Particles, Kwangju, Korea; 81 Hanyang University, Seoul, Korea; 82 Korea University, Seoul, Korea; 83 Seoul National University, Seoul, Korea; 84 University of Seoul, Seoul, Korea; 85 Sungkyunkwan University, Suwon, Korea; 86 Vilnius University, Vilnius, Lithuania; 87 National Centre for Particle Physics, Universiti Malaya, Kuala Lumpur, Malaysia; 88 Centro de Investigacion y de Estudios Avanzados del IPN, Mexico City, Mexico; 89 Universidad Iberoamericana, Mexico City, Mexico; 90 Benemerita Universidad Autonoma de Puebla, Puebla, Mexico; 91 Universidad Autónoma de San Luis Potosí, San Luis Potosi, Mexico; 92 University of Auckland, Auckland, New Zealand; 93 University of Canterbury, Christchurch, New Zealand; 94 National Centre for Physics, Quaid-I-Azam University, Islamabad, Pakistan; 95 National Centre for Nuclear Research, Swierk, Poland; 96 Institute of Experimental Physics, Faculty of Physics, University of Warsaw, Warsaw, Poland; 97 Laboratório de Instrumentação e Física Experimental de Partículas, Lisboa, Portugal; 98 Joint Institute for Nuclear Research, Dubna, Russia; 99 Petersburg Nuclear Physics Institute, Gatchina, (St. Petersburg), Russia; 100 Institute for Nuclear Research, Moscow, Russia; 101 Institute for Theoretical and Experimental Physics, Moscow, Russia; 102 Moscow Institute of Physics and Technology, Moscow, Russia; 103 National Research Nuclear University ‘Moscow Engineering Physics Institute’ (MEPhI), Moscow, Russia; 104 P.N. Lebedev Physical Institute, Moscow, Russia; 105 Skobeltsyn Institute of Nuclear Physics, Lomonosov Moscow State University, Moscow, Russia; 106 Novosibirsk State University (NSU), Novosibirsk, Russia; 107 State Research Center of Russian Federation, Institute for High Energy Physics, Protvino, Russia; 108 University of Belgrade, Faculty of Physics and Vinca Institute of Nuclear Sciences, Belgrade, Serbia; 109 Centro de Investigaciones Energéticas Medioambientales y Tecnológicas (CIEMAT), Madrid, Spain; 110 Universidad Autónoma de Madrid, Madrid, Spain; 111 Universidad de Oviedo, Oviedo, Spain; 112 Instituto de Física de Cantabria (IFCA), CSIC-Universidad de Cantabria, Santander, Spain; 113 CERN, European Organization for Nuclear Research, Geneva, Switzerland; 114 Paul Scherrer Institut, Villigen, Switzerland; 115 ETH Zurich - Institute for Particle Physics and Astrophysics (IPA), Zurich, Switzerland; 116 Universität Zürich, Zurich, Switzerland; 117 National Central University, Chung-Li, Taiwan; 118 National Taiwan University (NTU), Taipei, Taiwan; 119 Chulalongkorn University, Faculty of Science, Department of Physics, Bangkok, Thailand; 120 Çukurova University, Physics Department, Science and Art Faculty, Adana, Turkey; 121 Middle East Technical University, Physics Department, Ankara, Turkey; 122 Bogazici University, Istanbul, Turkey; 123 Istanbul Technical University, Istanbul, Turkey; 124 Institute for Scintillation Materials of National Academy of Science of Ukraine, Kharkov, Ukraine; 125 National Scientific Center, Kharkov Institute of Physics and Technology, Kharkov, Ukraine; 126 University of Bristol, Bristol, UK; 127 Rutherford Appleton Laboratory, Didcot, UK; 128 Imperial College, London, UK; 129 Brunel University, Uxbridge, UK; 130 Baylor University, Waco, USA; 131 Catholic University of America, Washington, DC, USA; 132 The University of Alabama, Tuscaloosa, USA; 133 Boston University, Boston, USA; 134 Brown University, Providence, USA; 135 University of California, Davis, Davis USA; 136 University of California, Los Angeles, USA; 137 University of California, Riverside, Riverside USA; 138 University of California, San Diego, La Jolla USA; 139 University of California, Santa Barbara-Department of Physics, Santa Barbara, USA; 140 California Institute of Technology, Pasadena, USA; 141 Carnegie Mellon University, Pittsburgh, USA; 142 University of Colorado Boulder, Boulder, USA; 143 Cornell University, Ithaca, USA; 144 Fermi National Accelerator Laboratory, Batavia, USA; 145 University of Florida, Gainesville, USA; 146 Florida International University, Miami, USA; 147 Florida State University, Tallahassee, USA; 148 Florida Institute of Technology, Melbourne, USA; 149 University of Illinois at Chicago (UIC), Chicago, USA; 150 The University of Iowa, Iowa City, USA; 151 Johns Hopkins University, Baltimore, USA; 152 The University of Kansas, Lawrence, USA; 153 Kansas State University, Manhattan, USA; 154 Lawrence Livermore National Laboratory, Livermore, USA; 155 University of Maryland, College Park, USA; 156 Massachusetts Institute of Technology, Cambridge, USA; 157 University of Minnesota, Minneapolis, USA; 158 University of Mississippi, Oxford, USA; 159 University of Nebraska-Lincoln, Lincoln, USA; 160 State University of New York at Buffalo, Buffalo, USA; 161 Northeastern University, Boston, USA; 162 Northwestern University, Evanston, USA; 163 University of Notre Dame, Notre Dame, USA; 164 The Ohio State University, Columbus, USA; 165 Princeton University, Princeton, USA; 166 University of Puerto Rico, Mayaguez, USA; 167 Purdue University, West Lafayette, USA; 168 Purdue University Northwest, Hammond, USA; 169 Rice University, Houston, USA; 170 University of Rochester, Rochester, USA; 171 The Rockefeller University, New York, USA; 172 Rutgers, The State University of New Jersey, Piscataway, USA; 173 University of Tennessee, Knoxville, USA; 174 Texas A & M University, College Station, USA; 175 Texas Tech University, Lubbock, USA; 176 Vanderbilt University, Nashville, USA; 177 University of Virginia, Charlottesville, USA; 178 Wayne State University, Detroit, USA; 179 University of Wisconsin-Madison, Madison, WI USA; 180 CERN, 1211 Geneva 23, Switzerland

## Abstract

Four-lepton production in proton-proton collisions, $\mathrm {p}\mathrm {p}\rightarrow (\mathrm{Z}/ \gamma ^*)(\mathrm{Z}/\gamma ^*) \rightarrow 4\ell$, where $\ell = \mathrm {e}$ or $\mu$, is studied at a center-of-mass energy of 13$\,\text {TeV}$ with the CMS detector at the LHC. The data sample corresponds to an integrated luminosity of 35.9$\,\text {fb}^{-1}$. The ZZ production cross section, $\sigma (\mathrm {p}\mathrm {p}\rightarrow \mathrm{Z}\mathrm{Z}) = 17.2 \pm 0.5\,\text {(stat)} \pm 0.7\,\text {(syst)} \pm 0.4\,\text {(theo)} \pm 0.4\,\text {(lumi)} \text { pb}$, measured using events with two opposite-sign, same-flavor lepton pairs produced in the mass region $60< m_{\ell ^+\ell ^-} < 120\,\text {GeV}$, is consistent with standard model predictions. Differential cross sections are measured and are well described by the theoretical predictions. The Z boson branching fraction to four leptons is measured to be $\mathcal {B}(\mathrm{Z}\rightarrow 4\ell ) = 4.83 _{-0.22}^{+0.23} (stat)_{-0.29}^{+0.32} (syst) \pm 0.08 (theo) \pm 0.12 (lumi) \times 10^{-6}$ for events with a four-lepton invariant mass in the range $80< m_{4\ell } < 100\,\text {GeV}$ and a dilepton mass $m_{\ell \ell } > 4\,\text {GeV}$ for all opposite-sign, same-flavor lepton pairs. The results agree with standard model predictions. The invariant mass distribution of the four-lepton system is used to set limits on anomalous ZZZ and ZZ$\gamma$ couplings at 95% confidence level: $-0.0012<f_4^\mathrm{Z}<0.0010$, $-0.0010<f_5^\mathrm{Z}<0.0013$, $-0.0012<f_4^{\gamma }<0.0013$, $-0.0012<f_5^{\gamma }< 0.0013$.

## Introduction

Measurements of diboson production at the CERN LHC allow precision tests of the standard model (SM). In the SM, $\mathrm{Z}\mathrm{Z}$ production proceeds mainly through quark-antiquark *t*- and *u*-channel scattering diagrams. In calculations at higher orders in quantum chromodynamics (QCD), gluon-gluon fusion also contributes via box diagrams with quark loops. There are no tree-level contributions to $\mathrm{Z}\mathrm{Z}$ production from triple gauge boson vertices in the SM. Anomalous triple gauge couplings (aTGC) could be induced by new physics models such as supersymmetry [1]. Nonzero aTGCs may be parametrized using an effective Lagrangian as in Ref. [2]. In this formalism, two $\mathrm{Z} \mathrm{Z} \mathrm{Z}$ and two $\mathrm{Z} \mathrm{Z} \gamma$ couplings are allowed by electromagnetic gauge invariance and Lorentz invariance for on-shell $\mathrm{Z}$ bosons. These are described by two CP-violating ($f_4^{\mathrm {V}}$) and two CP-conserving ($f_5^{\mathrm {V}}$) parameters, where ${\mathrm {V}} = \mathrm{Z}$ or $\gamma$.

Previous measurements of the ZZ production cross section by the CMS Collaboration were performed for pairs of on-shell $\mathrm{Z}$ bosons, produced in the dilepton mass range 60–120$\,\text {GeV}$  [3–6]. These measurements were made with data sets corresponding to integrated luminosities of 5.1$\,\text {fb}^{-1}$ at $\sqrt{s} = 7\,\text {TeV}$ and 19.6$\,\text {fb}^{-1}$ at $\sqrt{s} = 8\,\text {TeV}$ in the $\mathrm{Z}\mathrm{Z}\rightarrow 2\ell 2\ell ^{\prime \prime }$ and $\mathrm{Z}\mathrm{Z}\rightarrow 2\ell 2\nu$ decay channels, where $\ell = \mathrm {e}$ or $\mathrm {\mu }$ and $\ell ^{\prime \prime } = \mathrm {e}, \mathrm {\mu }$, or $\mathrm {\tau }$, and with an integrated luminosity of 2.6$\,\text {fb}^{-1}$ at $\sqrt{s} = 13\,\text {TeV}$ in the $\mathrm{Z}\mathrm{Z} \rightarrow 2\ell 2\ell '$ decay channel, where $\ell ' = \mathrm {e}$ or $\mathrm {\mu }$. All of them agree with SM predictions. The ATLAS Collaboration produced similar results at $\sqrt{s} = 7$, 8, and 13$\,\text {TeV}$  [7–10], which also agree with the SM. These measurements are important for testing predictions that were recently made available at next-to-next-to-leading order (NNLO) in QCD [11]. Comparing these predictions with data at a range of center-of-mass energies provides information about the electroweak gauge sector of the SM. Because the uncertainty of the CMS measurement at $\sqrt{s} = 13\,\text {TeV}$ [6] was dominated by the statistical uncertainty of the observed data, repeating and extending the measurement with a larger sample of proton-proton collision data at $\sqrt{s} = 13\,\text {TeV}$ improves the precision of the results.

The most stringent previous limits on $\mathrm{Z}\mathrm{Z}\mathrm{Z}$ and $\mathrm{Z}\mathrm{Z}\gamma$ aTGCs from CMS were set using the 7 and $8\,\text {TeV}$ data samples: $-0.0022<f_4^\mathrm{Z}<0.0026$, $-0.0023<f_5^\mathrm{Z}<0.0023$, $-0.0029<f_4^{\gamma }<0.0026$, and $-0.0026<f_5^{\gamma }<0.0027$ at 95% confidence level (CL) [4, 5]. Similar limits were obtained by the ATLAS Collaboration [12], who also recently produced limits using $13\,\text {TeV}$ data [10].

Extending the dilepton mass range to lower values allows measurements of $\left( \mathrm{Z}/\gamma ^{*}\right) \left( \mathrm{Z}/\gamma ^{*}\right)$ production, where $\mathrm{Z}$ indicates an on-shell $\mathrm{Z}$ boson or an off-shell $\mathrm{Z}^{*}$ boson. The resulting sample includes Higgs boson events in the $\mathrm{H} \rightarrow \mathrm{Z}\mathrm{Z}^{*} \rightarrow 2\ell 2\ell '$ channel, and rare decays of a single $\mathrm{Z}$ boson to four leptons. The $\mathrm{Z}\rightarrow \ell ^+\ell ^-\gamma ^{*} \rightarrow 2\ell 2\ell '$ decay was studied in detail at LEP [13] and was observed in pp collisions by CMS [6, 14] and ATLAS [15]. Although the branching fraction for this decay is orders of magnitude smaller than that for the $\mathrm{Z}\rightarrow \ell ^+\ell ^-$ decay, the precisely known mass of the $\mathrm{Z}$ boson makes the four-lepton mode useful for calibrating mass measurements of the nearby Higgs boson resonance.

This paper reports a study of four-lepton production ($\mathrm {p}\mathrm {p} \rightarrow 2\ell 2\ell '$, where $2\ell$ and $2\ell '$ indicate opposite-sign pairs of electrons or muons) at $\sqrt{s} = 13\,\text {TeV}$ with a data set corresponding to an integrated luminosity of $35.9 \pm 0.9$ $\,\text {fb}^{-1}$ recorded in 2016. Cross sections are measured for nonresonant production of pairs of $\mathrm{Z}$ bosons, $\mathrm {p}\mathrm {p} \rightarrow \mathrm{Z}\mathrm{Z}$, where both $\mathrm{Z}$ bosons are produced on-shell, defined as the mass range 60–120$\,\text {GeV}$, and resonant $\mathrm {p}\mathrm {p} \rightarrow \mathrm{Z}\rightarrow 4\ell$ production. Detailed discussion of resonant Higgs boson production decaying to $\mathrm{Z}\mathrm{Z}^*$, is beyond the scope of this paper and may be found in Ref. [16].

## The CMS detector

A detailed description of the CMS detector, together with a definition of the coordinate system used and the relevant kinematic variables, can be found in Ref. [17].

The central feature of the CMS apparatus is a superconducting solenoid of 6$\text { m}$ internal diameter, providing a magnetic field of 3.8$\text { T}$. Within the solenoid volume are a silicon pixel and strip tracker, a lead tungstate crystal electromagnetic calorimeter (ECAL), and a brass and scintillator hadron calorimeter, which provide coverage in pseudorapidity $| \eta | < 1.479$ in a cylindrical barrel and $1.479< | \eta | < 3.0$ in two endcap regions. Forward calorimeters extend the coverage provided by the barrel and endcap detectors to $|\eta | < 5.0$. Muons are measured in gas-ionization detectors embedded in the steel flux-return yoke outside the solenoid in the range $|\eta | < 2.4$, with detection planes made using three technologies: drift tubes, cathode strip chambers, and resistive plate chambers.

Electron momenta are estimated by combining energy measurements in the ECAL with momentum measurements in the tracker. The momentum resolution for electrons with transverse momentum $p_{\mathrm {T}} \approx 45\,\text {GeV}$ from $\mathrm{Z}\rightarrow \mathrm {e}^+\mathrm {e}^-$ decays ranges from 1.7% for nonshowering electrons in the barrel region to 4.5% for showering electrons in the endcaps [18]. Matching muons to tracks identified in the silicon tracker results in a $p_{\mathrm {T}}$ resolution for muons with $20<p_{\mathrm {T}} < 100\,\text {GeV}$ of 1.3–2.0% in the barrel and better than 6% in the endcaps. The $p_{\mathrm {T}}$ resolution in the barrel is better than 10% for muons with $p_{\mathrm {T}}$ up to 1$\,\text {TeV}$  [19].

## Signal and background simulation

Signal events are generated with powheg  2.0 [20–24] at next-to-leading order (NLO) in QCD for quark-antiquark processes and leading order (LO) for quark-gluon processes. This includes $\mathrm{Z}\mathrm{Z}$, $\mathrm{Z}\gamma ^*$, $\mathrm{Z}$, and $\gamma ^*\gamma ^*$ production with a constraint of $m_{\ell \ell '} > 4\,\text {GeV}$ applied to all pairs of oppositely charged leptons at the generator level to avoid infrared divergences. The $\mathrm{g} \mathrm{g} \rightarrow \mathrm{Z}\mathrm{Z}$ process is simulated at LO with mcfm  v7.0 [25]. These samples are scaled to correspond to cross sections calculated at NNLO in QCD for $\mathrm{q}\overline{\mathrm{q}}\rightarrow \mathrm{Z}\mathrm{Z}$ [11] (a scaling *K* factor of 1.1) and at NLO in QCD for $\mathrm{g} \mathrm{g} \rightarrow \mathrm{Z}\mathrm{Z}$ [26] (*K* factor of 1.7). The $\mathrm{g} \mathrm{g} \rightarrow \mathrm{Z}\mathrm{Z}$ process is calculated to $\mathcal {O}\left( \alpha _s^3\right)$, where $\alpha _s$ is the strong coupling constant, while the other contributing processes are calculated to $\mathcal {O}\left( \alpha _s^2\right)$; this higher-order correction is included because the effect is known to be large [26]. Electroweak $\mathrm{Z}\mathrm{Z}$ production in association with two jets is generated with Phantom v1.2.8 [27].

A sample of Higgs boson events is produced in the gluon-gluon fusion process at NLO with powheg. The Higgs boson decay is modeled with jhugen 3.1.8 [28–30]. Its cross section is scaled to the NNLO prediction with a *K* factor of 1.7 [26].

Samples for background processes containing four prompt leptons in the final state, $\mathrm{t}\overline{\mathrm{t}} \mathrm{Z}$ and $\mathrm {W}\mathrm {W}\mathrm{Z}$ production, are produced with MadGraph 5_amc@nlo v2.3.3 [31]. The $\mathrm{q}\overline{\mathrm{q}}\rightarrow \mathrm {W}\mathrm{Z}$ process is generated with powheg.

Samples with aTGC contributions included are generated at LO with sherpa v2.1.1 [32]. Distributions from the sherpa samples are normalized such that the total yield of the SM sample is the same as that of the powheg sample.

The pythia  v8.175 [23, 33, 34] package is used for parton showering, hadronization, and the underlying event simulation, with parameters set by the CUETP8M1 tune [35], for all samples except the samples generated with sherpa, which performs these functions itself. The NNPDF 3.0 [36] set is used as the default set of parton distribution functions (PDFs). For all simulated event samples, the PDFs are calculated to the same order in QCD as the process in the sample.

The detector response is simulated using a detailed description of the CMS detector implemented with the Geant4 package [37]. The event reconstruction is performed with the same algorithms used for data. The simulated samples include additional interactions per bunch crossing, referred to as pileup. The simulated events are weighted so that the pileup distribution matches the data, with an average of about 27 interactions per bunch crossing.

## Event reconstruction

All long-lived particles—electrons, muons, photons, and charged and neutral hadrons—in each collision event are identified and reconstructed with the CMS particle-flow (PF) algorithm [38] from a combination of the signals from all subdetectors. Reconstructed electrons [18] and muons [19] are considered candidates for inclusion in four-lepton final states if they have $p_{\mathrm {T}} ^\mathrm {e}> 7\,\text {GeV}$ and $|\eta ^\mathrm {e} | < 2.5$ or $p_{\mathrm {T}} ^\mathrm {\mu }> 5\,\text {GeV}$ and $|\eta ^\mathrm {\mu } | < 2.4$.

Lepton candidates are also required to originate from the event vertex, defined as the reconstructed proton-proton interaction vertex with the largest value of summed physics object $p_{\mathrm {T}} ^2$. The physics objects used in the event vertex definition are the objects returned by a jet finding algorithm [39, 40] applied to all charged tracks associated with the vertex, plus the corresponding associated missing transverse momentum [41]. The distance of closest approach between each lepton track and the event vertex is required to be less than 0.5$\text { cm}$ in the plane transverse to the beam axis, and less than 1$\text { cm}$ in the direction along the beam axis. Furthermore, the significance of the three-dimensional impact parameter relative to the event vertex, $\mathrm {SIP_{3D}}$, is required to satisfy $\mathrm {SIP_{3D}} \equiv | \mathrm {IP} / \sigma _\mathrm {IP} | < 10$ for each lepton, where $\mathrm {IP}$ is the distance of closest approach of each lepton track to the event vertex and $\sigma _\mathrm {IP}$ is its associated uncertainty.

Lepton candidates are required to be isolated from other particles in the event. The relative isolation is defined as

1 $$\begin{aligned} R_\text {iso} = \bigg [ \sum _{\begin{array}{c} \text {charged} \\ \text {hadrons} \end{array}} \!\! p_{\mathrm {T}} \, + \, \max \big (0, \sum _{\begin{array}{c} \text {neutral} \\ \text {hadrons} \end{array}} \!\! p_{\mathrm {T}} \, + \, \sum _{\text {photons}} \!\! p_{\mathrm {T}} \, - \, p_{\mathrm {T}} ^\mathrm {PU} \big )\bigg ] \bigg / p_{\mathrm {T}} ^{\ell }, \end{aligned}$$

where the sums run over the charged and neutral hadrons and photons identified by the PF algorithm, in a cone defined by $\Delta R \equiv \sqrt{\smash [b]{\left( \Delta \eta \right) ^2 + \left( \Delta \phi \right) ^2}} < 0.3$ around the lepton trajectory. Here $\phi$ is the azimuthal angle in radians. To minimize the contribution of charged particles from pileup to the isolation calculation, charged hadrons are included only if they originate from the event vertex. The contribution of neutral particles from pileup is $p_{\mathrm {T}} ^\mathrm {PU}$. For electrons, $p_{\mathrm {T}} ^\mathrm {PU}$ is evaluated with the “jet area” method described in Ref. [42]; for muons, it is taken to be half the sum of the $p_{\mathrm {T}}$ of all charged particles in the cone originating from pileup vertices. The factor one-half accounts for the expected ratio of charged to neutral particle energy in hadronic interactions. A lepton is considered isolated if $R_\text {iso} < 0.35$.

The lepton reconstruction, identification, and isolation efficiencies are measured with a “tag-and-probe” technique [43] applied to a sample of $\mathrm{Z}\rightarrow \ell ^+\ell ^-$ data events. The measurements are performed in several bins of $p_{\mathrm {T}} ^{\ell }$ and $|\eta ^\ell |$. The electron reconstruction and selection efficiency in the ECAL barrel (endcaps) varies from about 85% (77%) at $p_{\mathrm {T}} ^{\mathrm {e}} \approx 10\,\text {GeV}$ to about 95% (89%) for $p_{\mathrm {T}} ^{\mathrm {e}} \ge 20\,\text {GeV}$, while in the barrel-endcap transition region this efficiency is about 85% averaged over all electrons with $p_{\mathrm {T}} ^{\mathrm {e}} > 7\,\text {GeV}$. The muons are reconstructed and identified with efficiencies above ${\sim }98\%$ within $|\eta ^{\mathrm {\mu }} | < 2.4$.

## Event selection

The primary triggers for this analysis require the presence of a pair of loosely isolated leptons of the same or different flavors [44]. The highest $p_{\mathrm {T}}$ lepton must have $p_{\mathrm {T}} ^\ell > 17\,\text {GeV}$, and the subleading lepton must have $p_{\mathrm {T}} ^\mathrm {e}> 12\,\text {GeV}$ if it is an electron or $p_{\mathrm {T}} ^\mathrm {\mu }> 8\,\text {GeV}$ if it is a muon. The tracks of the triggering leptons are required to originate within 2 mm of each other in the plane transverse to the beam axis. Triggers requiring a triplet of lower-$p_{\mathrm {T}}$ leptons with no isolation criterion, or a single high-$p_{\mathrm {T}}$ electron or muon, are also used. An event is used if it passes any trigger regardless of the decay channel. The total trigger efficiency for events within the acceptance of this analysis is greater than 98%.

The four-lepton candidate selections are based on those used in Ref. [45]. A signal event must contain at least two $\mathrm{Z}/\gamma ^{*}$ candidates, each formed from an oppositely charged pair of isolated electron candidates or muon candidates. Among the four leptons, the highest $p_{\mathrm {T}}$ lepton must have $p_{\mathrm {T}} > 20\,\text {GeV}$, and the second-highest $p_{\mathrm {T}}$ lepton must have $p_{\mathrm {T}} ^\mathrm {e}> 12\,\text {GeV}$ if it is an electron or $p_{\mathrm {T}} ^\mathrm {\mu }> 10\,\text {GeV}$ if it is a muon. All leptons are required to be separated from each other by $\Delta R \left( \ell _1, \ell _2 \right) > 0.02$, and electrons are required to be separated from muons by $\Delta R \left( \mathrm {e}, \mu \right) > 0.05$.

Within each event, all permutations of leptons giving a valid pair of $\mathrm{Z}/\gamma ^{*}$ candidates are considered separately. Within each $4\ell$ candidate, the dilepton candidate with an invariant mass closest to 91.2$\,\text {GeV}$, taken as the nominal $\mathrm{Z}$ boson mass [46], is denoted $\mathrm{Z}_1$ and is required to have a mass greater than 40$\,\text {GeV}$. The other dilepton candidate is denoted $\mathrm{Z}_2$. Both $m_{\mathrm{Z}_1}$ and $m_{\mathrm{Z}_2}$ are required to be less than 120$\,\text {GeV}$. All pairs of oppositely charged leptons in the $4\ell$ candidate are required to have $m_{\ell \ell '} > 4\,\text {GeV}$ regardless of their flavor.

If multiple $4\ell$ candidates within an event pass all selections, the one with $m_{\mathrm{Z}_1}$ closest to the nominal $\mathrm{Z}$ boson mass is chosen. In the rare case of further ambiguity, which may arise in less than 0.5% of events when five or more passing lepton candidates are found, the $\mathrm{Z}_2$ candidate that maximizes the scalar $p_{\mathrm {T}}$ sum of the four leptons is chosen.

Additional requirements are applied to select events for measurements of specific processes. The $\mathrm {p}\mathrm {p} \rightarrow \mathrm{Z}\mathrm{Z}$ cross section is measured using events where both $m_{\mathrm{Z}_1}$ and $m_{\mathrm{Z}_2}$ are greater than 60$\,\text {GeV}$. The $\mathrm{Z}\rightarrow 4\ell$ branching fraction is measured using events with $80< m_{4\ell } < 100\,\text {GeV}$, a range chosen to retain most of the decays in the resonance while removing most other processes with four-lepton final states. Decays of the $\mathrm{Z}$ bosons to $\tau$ leptons with subsequent decays to electrons and muons are heavily suppressed by requirements on lepton $p_{\mathrm {T}}$, and the contribution of such events is less than 0.5% of the total $\mathrm{Z}\mathrm{Z}$ yield. If these events pass the selection requirements of the analysis, they are considered signal, while they are not considered at generator level in the cross section unfolding procedure. Thus, the correction for possible $\tau$ decays is included in the efficiency calculation.

## Background estimate

The major background contributions arise from $\mathrm{Z}$ boson and $\mathrm {W}\mathrm{Z}$ diboson production in association with jets and from $\mathrm{t}\overline{\mathrm{t}}$ production. In all these cases, particles from jet fragmentation satisfy both lepton identification and isolation criteria, and are thus misidentified as signal leptons.

The probability for such objects to be selected is measured from a sample of $\mathrm{Z}+ \ell _\text {candidate}$ events, where $\mathrm{Z}$ denotes a pair of oppositely charged, same-flavor leptons that pass all analysis requirements and satisfy $| m_{\ell ^+\ell ^-} - m_{\mathrm{Z}} | < 10\,\text {GeV}$, where $m_\mathrm{Z}$ is the nominal $\mathrm{Z}$ boson mass. Each event in this sample must have exactly one additional object $\ell _\text {candidate}$ that passes relaxed identification requirements with no isolation requirements applied. The misidentification probability for each lepton flavor, measured in bins of lepton candidate $p_{\mathrm {T}}$ and $\eta$, is defined as the ratio of the number of candidates that pass the final isolation and identification requirements to the total number in the sample. The number of $\mathrm{Z}+ \ell _\text {candidate}$ events is corrected for the contamination from $\mathrm {W}\mathrm{Z}$ production and $\mathrm{Z}\mathrm{Z}$ production in which one lepton is not reconstructed. These events have a third genuine, isolated lepton that must be excluded from the misidentification probability calculation. The WZ contamination is suppressed by requiring the missing transverse momentum $p_{\mathrm {T}} ^\text {miss}$ to be below 25$\,\text {GeV}$. The $p_{\mathrm {T}} ^\text {miss}$ is defined as the magnitude of the missing transverse momentum vector ${\vec p}_{\mathrm {T}}^{\text {miss}}$, the projection onto the plane transverse to the beams of the negative vector sum of the momenta of all reconstructed PF candidates in the event, corrected for the jet energy scale. Additionally, the transverse mass calculated with ${\vec p}_{\mathrm {T}}^{\text {miss}}$ and the ${\vec {p}}_{\mathrm {T}}$ of $\ell _\text {candidate}$, $m_{\mathrm {T}} \equiv \sqrt{\smash [b]{(p_{\mathrm {T}} ^\ell + p_{\mathrm {T}} ^\text {miss})^2 - ({\vec {p}}_{\mathrm {T}} ^{\ell } + {\vec p}_{\mathrm {T}}^{\text {miss}})^2}}$, is required to be less than 30$\,\text {GeV}$. The residual contribution of $\mathrm {W}\mathrm{Z}$ and $\mathrm{Z}\mathrm{Z}$ events, which may be up to a few percent of the events with $\ell _\text {candidate}$ passing all selection criteria, is estimated from simulation and subtracted.

To account for all sources of background events, two control samples are used to estimate the number of background events in the signal regions. Both are defined to contain events with a dilepton candidate satisfying all requirements ($\mathrm{Z}_1$) and two additional lepton candidates $\ell ^{\prime +}\ell ^{\prime -}$. In one control sample, enriched in $\mathrm {W}\mathrm{Z}$ events, one $\ell ^{\prime }$ candidate is required to satisfy the full identification and isolation criteria and the other must fail the full criteria and instead satisfy only the relaxed ones; in the other, enriched in $\mathrm{Z}$+jets events, both $\ell ^{\prime }$ candidates must satisfy the relaxed criteria, but fail the full criteria. The additional leptons must have opposite charge and the same flavor ($\mathrm {e}^{\pm }\mathrm {e}^{\mp }, \mathrm {\mu }^{\pm }\mathrm {\mu }^{\mp }$). From this set of events, the expected number of background events in the signal region, denoted “$\mathrm{Z}+ \text {X}$” in the figures, is obtained by scaling the number of observed $\mathrm{Z}_1+\ell ^{\prime +}\ell ^{\prime -}$ events by the misidentification probability for each lepton failing the selection. It is found to be approximately 4% of the total expected yield. The procedure is described in more detail in Ref. [45].

In addition to these nonprompt backgrounds, $\mathrm{t}\overline{\mathrm{t}} \mathrm{Z}$ and $\mathrm {W}\mathrm {W}\mathrm{Z}$ processes contribute a smaller number of events with four prompt leptons, which is estimated from simulated samples to be around 1% of the expected $\mathrm{Z}\mathrm{Z} \rightarrow 4\ell$ yield. In the $\mathrm{Z}\rightarrow 4\ell$ selection, the contribution from these backgrounds is negligible. The total background contributions to the $\mathrm{Z}\rightarrow 4\ell$ and $\mathrm{Z}\mathrm{Z}\rightarrow 4\ell$ signal regions are summarized in Section 8.

## Systematic uncertainties

The major sources of systematic uncertainty and their effect on the measured cross sections are summarized in Table 1. In both data and simulated event samples, trigger efficiencies are evaluated with a tag-and-probe technique. The ratio of data to simulation is applied to simulated events, and the size of the resulting change in expected yield is taken as the uncertainty in the determination of the trigger efficiency. This uncertainty is around 2% of the final estimated yield. For $\mathrm{Z}\rightarrow 4\mathrm {e}$ events, the uncertainty increases to 4%.

**Table 1:** The contributions of each source of systematic uncertainty in the cross section measurements. The integrated luminosity uncertainty, and the PDF and scale uncertainties, are considered separately. All other uncertainties are added in quadrature into a single systematic uncertainty. Uncertainties that vary by decay channel are listed as a range

Uncertainty	$\mathrm{Z}\rightarrow 4\ell$ (%)	$\mathrm{Z}\mathrm{Z}\rightarrow 4\ell$ (%)
Lepton efficiency	6–10	2–6
Trigger efficiency	2–4	2
Statistical (simulation)	1–2	0.5
Background	0.6–1.3	0.5–1
Pileup	1–2	1
PDF	1	1
$\mu _\mathrm {R}$, $\mu _\mathrm {F}$	1	1
Integrated luminosity	2.5	2.5

The lepton identification, isolation, and track reconstruction efficiencies in simulation are corrected with scaling factors derived with a tag-and-probe method and applied as a function of lepton $p_{\mathrm {T}}$ and $\eta$. To estimate the uncertainties associated with the tag-and-probe technique, the total yield is recomputed with the scaling factors varied up and down by the tag-and-probe fit uncertainties. The uncertainties associated with lepton efficiency in the $\mathrm{Z}\mathrm{Z} \rightarrow 4\ell$ ($\mathrm{Z}\rightarrow 4\ell$) signal regions are found to be 6(10)% in the $4\mathrm {e}$, 3(6)% in the $2\mathrm {e}2\mu$, and 2(7)% in the $4\mu$ final states. These uncertainties are higher for $\mathrm{Z}\rightarrow 4\ell$ events because the leptons generally have lower $p_{\mathrm {T}}$, and the samples used in the tag-and-probe method have fewer events and more contamination from nonprompt leptons in this low-$p_{\mathrm {T}}$ region.

Uncertainties due to the effect of factorization ($\mu _\mathrm {F}$) and renormalization ($\mu _\mathrm {R}$) scale choices on the $\mathrm{Z}\mathrm{Z} \rightarrow 4\ell$ acceptance are evaluated with powheg and mcfm by varying the scales up and down by a factor of two with respect to the default values $\mu _\mathrm {F} = \mu _\mathrm {R} = m_{\mathrm{Z}\mathrm{Z}}$. All combinations are considered except those in which $\mu _\mathrm {F}$ and $\mu _\mathrm {R}$ differ by a factor of four. Parametric uncertainties (PDF$+ \alpha _s$) are evaluated according to the pdf4lhc prescription [47] in the acceptance calculation, and with NNPDF3.0 in the cross section calculations. An additional theoretical uncertainty arises from scaling the powheg $\mathrm{q}\overline{\mathrm{q}}\rightarrow \mathrm{Z}\mathrm{Z}$ simulated sample from its NLO cross section to the NNLO prediction, and the mcfm $\mathrm{g} \mathrm{g} \rightarrow \mathrm{Z}\mathrm{Z}$ samples from their LO cross sections to the NLO predictions. The change in the acceptance corresponding to this scaling procedure is found to be 1.1%. All these theoretical uncertainties are added in quadrature.

The largest uncertainty in the estimated background yield arises from differences in sample composition between the $\mathrm{Z}+ \ell _\text {candidate}$ control sample used to calculate the lepton misidentification probability and the $\mathrm{Z}+ \ell ^+\ell ^-$ control sample. A further uncertainty arises from the limited number of events in the $\mathrm{Z}+ \ell _\text {candidate}$ sample. A systematic uncertainty of 40% is applied to the lepton misidentification probability to cover both effects. The size of this uncertainty varies by channel, but is less than 1% of the total expected yield.

The uncertainty in the integrated luminosity of the data sample is 2.5% [48].

## Cross section measurements

**Fig. 1:**
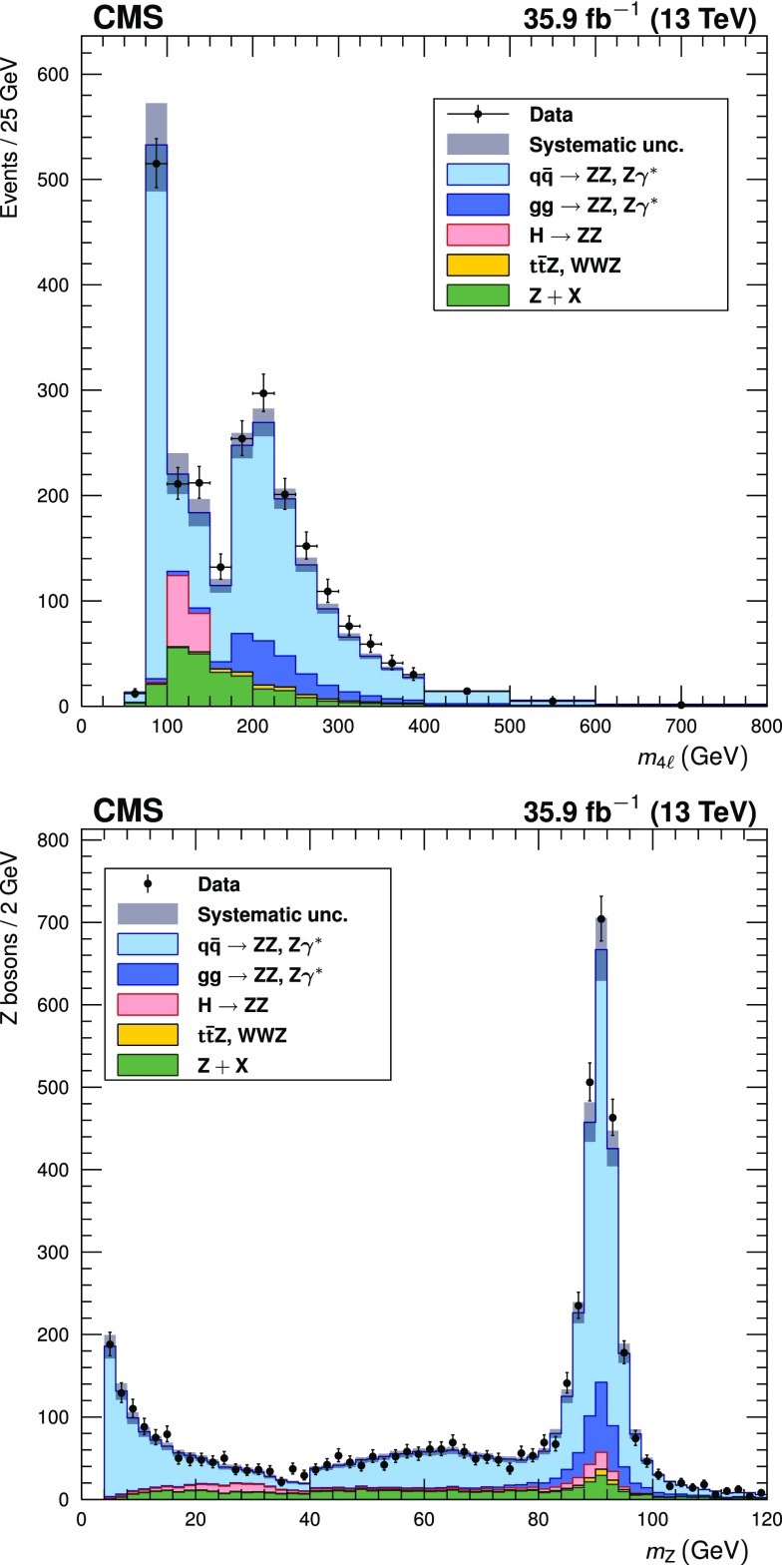
Distributions of (upper) the four-lepton invariant mass $m_{4\ell }$ and (lower) the dilepton invariant mass of all $\mathrm{Z}/\gamma ^*$ bosons in selected four-lepton events. Both selected dilepton candidates are included in each event. In the $m_{4\ell }$ distribution, bin contents are normalized to a bin width of 25$\,\text {GeV}$; horizontal bars on the data points show the range of the corresponding bin. Points represent the data, while filled histograms represent the SM prediction and background estimate. Vertical bars on the data points show their statistical uncertainty. Shaded grey regions around the predicted yield represent combined statistical, systematic, theoretical, and integrated luminosity uncertainties

**Fig. 2:**
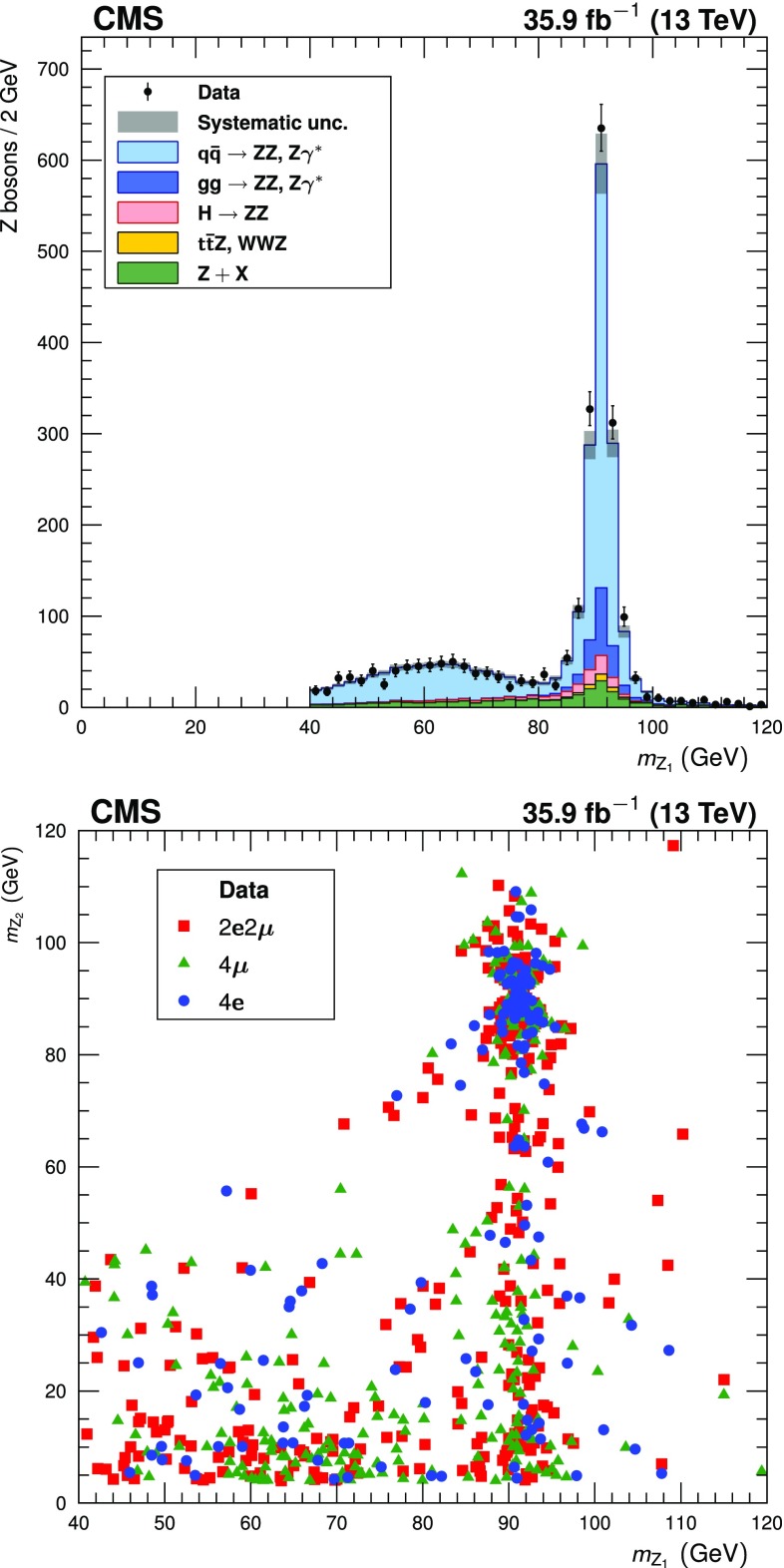
(Upper): the distribution of the reconstructed mass of $\mathrm{Z}_1$, the dilepton candidate closer to the nominal $\mathrm{Z}$ boson mass. Points represent the data, while filled histograms represent the SM prediction and background estimate. Vertical bars on the data points show their statistical uncertainty. Shaded grey regions around the predicted yield represent combined statistical, systematic, theoretical, and integrated luminosity uncertainties. (Lower): the reconstructed $m_{\mathrm{Z}_2}$ plotted against the reconstructed $m_{\mathrm{Z}_1}$ in data events, with distinctive markers for each final state. For readability, only every fourth event is plotted

The distributions of the four-lepton mass and the masses of the $\mathrm{Z}_1$ and $\mathrm{Z}_2$ candidates are shown in Fig. 1. The expected distributions describe the data well within uncertainties. The SM predictions include nonresonant $\mathrm{Z}\mathrm{Z}$ predictions, production of the SM Higgs boson with mass 125$\,\text {GeV}$  [49], and resonant $\mathrm{Z}\rightarrow 4\ell$ production. The backgrounds estimated from data and simulation are also shown. The reconstructed invariant mass of the $\mathrm{Z}_1$ candidates, and a scatter plot showing the correlation between $m_{\mathrm{Z}_2}$ and $m_{\mathrm{Z}_1}$ in data events, are shown in Fig. 2. In the scatter plot, clusters of events corresponding to $\mathrm{Z}\mathrm{Z}\rightarrow 4\ell$, $\mathrm{Z}\gamma ^*\rightarrow 4\ell$, and $\mathrm{Z}\rightarrow 4\ell$ production can be seen.

**Fig. 3:**
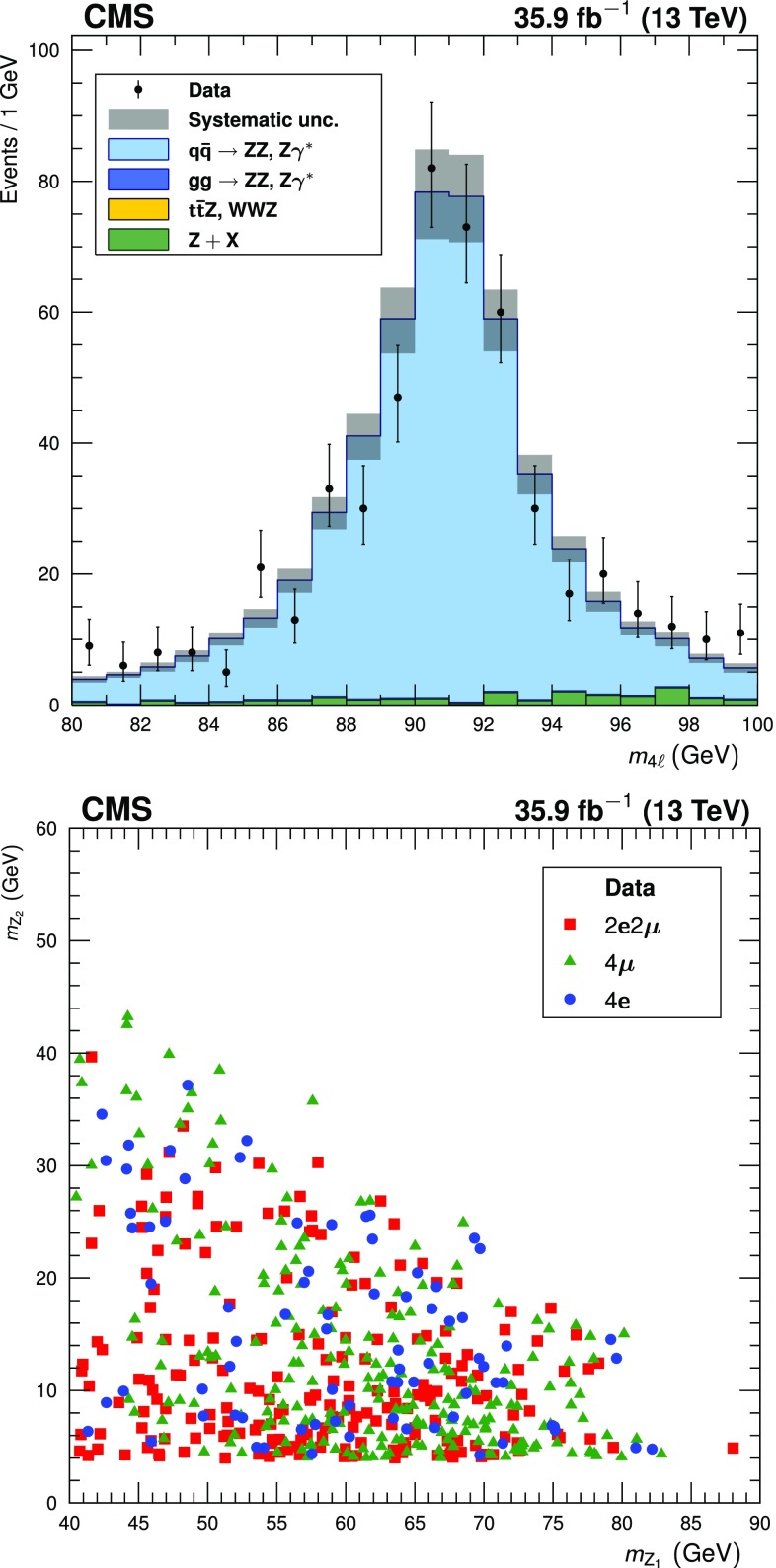
(Upper): the distribution of the reconstructed four-lepton mass $m_{4\ell }$ for events selected with $80< m_{4\ell } < 100\,\text {GeV}$. Points represent the data, while filled histograms represent the SM prediction and background estimate. Vertical bars on the data points show their statistical uncertainty. Shaded grey regions around the predicted yield represent combined statistical, systematic, theoretical, and integrated luminosity uncertainties. (Lower): the reconstructed $m_{\mathrm{Z}_2}$ plotted against the reconstructed $m_{\mathrm{Z}_1}$ for all data events selected with $m_{4\ell }$ between 80 and 100$\,\text {GeV}$, with distinctive markers for each final state

The four-lepton invariant mass distribution below 100$\,\text {GeV}$ is shown in Fig. 3 (upper). Figure 3 (lower) shows $m_{\mathrm{Z}_2}$ plotted against $m_{\mathrm{Z}_1}$ for events with $m_{4\ell }$ between 80 and 100$\,\text {GeV}$, and the observed and expected event yields in this mass region are given in Table 2. The yield of events in the $4\mathrm {e}$ final state is significantly lower than in the $4\mathrm {\mu }$ final state because minimum $p_{\mathrm {T}}$ thresholds are higher for electrons than for muons, and inefficiencies in the detection of low-$p_{\mathrm {T}}$ leptons affect electrons more strongly than they affect muons.

**Table 2:** The observed and expected yields of four-lepton events in the mass region $80< m_{4\ell } < 100\,\text {GeV}$ and estimated yields of background events, shown for each final state and summed in the total expected yield. The first uncertainty is statistical, the second one is systematic. The systematic uncertainties do not include the uncertainty in the integrated luminosity

Final	Expected	Background	Total	Observed
state	$N_{4\ell }$		expected	
$4\mathrm {\mu }$	$224 \pm 1 \pm 16$	$7 \pm 1 \pm 2$	$231 \pm 2 \pm 17$	225
$2\mathrm {e}2\mathrm {\mu }$	$207 \pm 1 \pm 14$	$9 \pm 1 \pm 2$	$216 \pm 2 \pm 14$	206
$4\mathrm {e}$	$68 \pm 1 \pm 8$	$4 \pm 1 \pm 2$	$72 \pm 1 \pm 8$	78
Total	$499 \pm 2 \pm 32$	$19 \pm 2 \pm 5$	$518 \pm 3 \pm 33$	509

**Fig. 4:**
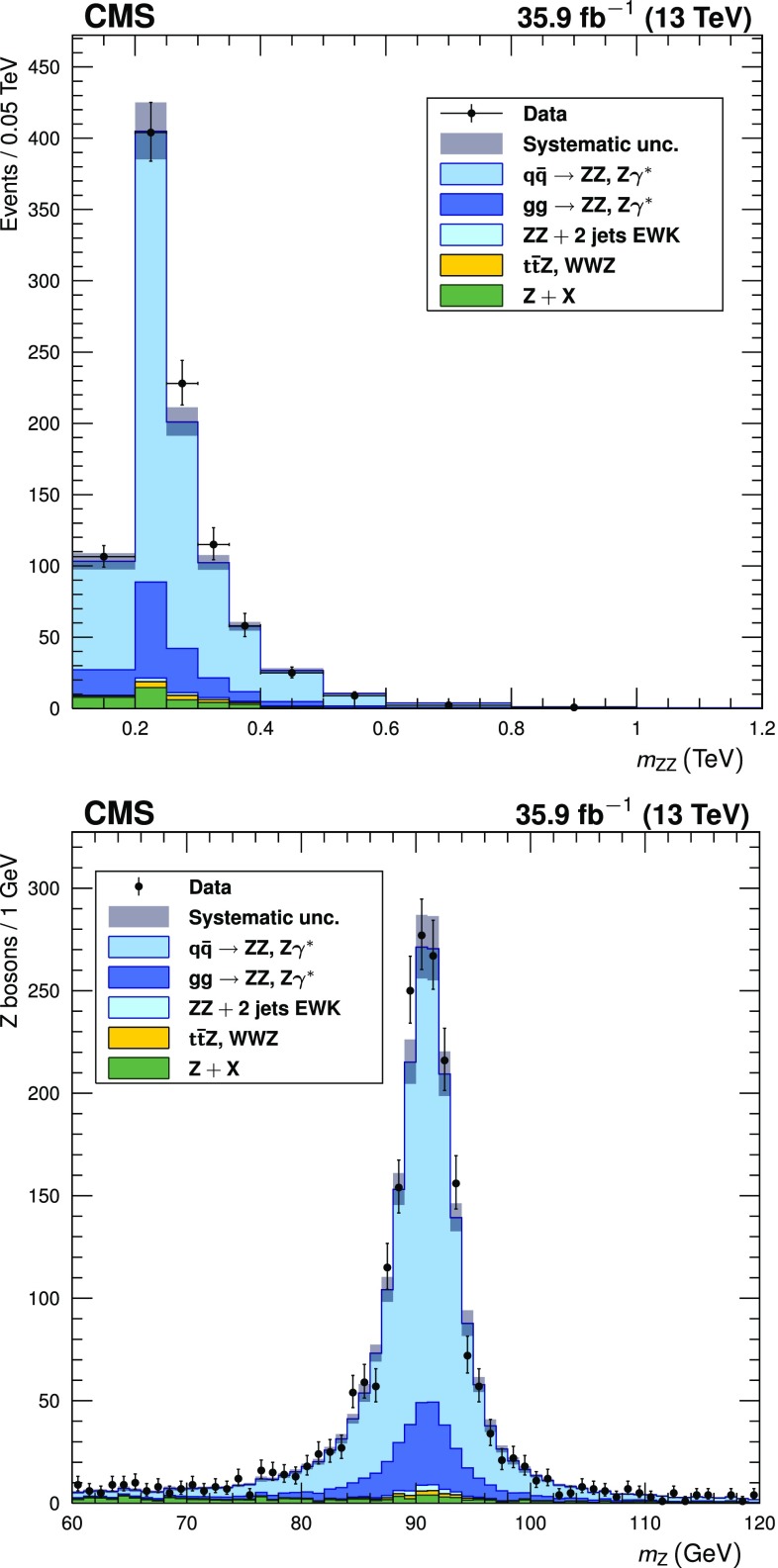
Distributions of (upper) the four-lepton invariant mass $m_{\mathrm{Z}\mathrm{Z}}$ and (lower) dilepton candidate mass for four-lepton events selected with both $\mathrm{Z}$ bosons on-shell. Points represent the data, while filled histograms represent the SM prediction and background estimate. Vertical bars on the data points show their statistical uncertainty. Shaded grey regions around the predicted yield represent combined statistical, systematic, theoretical, and integrated luminosity uncertainties. In the $m_{\mathrm{Z}\mathrm{Z}}$ distribution, bin contents are normalized to the bin widths, using a unit bin size of 50$\,\text {GeV}$; horizontal bars on the data points show the range of the corresponding bin

The reconstructed four-lepton invariant mass is shown in Fig. 4 (upper) for events with two on-shell $\mathrm{Z}$ bosons. Figure 4 (lower) shows the invariant mass distribution for all $\mathrm{Z}$ boson candidates in these events. The corresponding observed and expected yields are given in Table 3.

**Table 3:** The observed and expected yields of $\mathrm{Z}\mathrm{Z}$ events, and estimated yields of background events, shown for each final state and summed in the total expected yield. The first uncertainty is statistical, the second one is systematic. The systematic uncertainties do not include the uncertainty in the integrated luminosity

Decay	Expected	Background	Total	Observed
channel	$N_{4\ell }$		expected	
$4\mathrm {\mu }$	$301 \pm 2 \pm 9$	$10 \pm 1 \pm 2$	$311 \pm 2 \pm 9$	335
$2\mathrm {e}2\mathrm {\mu }$	$503 \pm 2 \pm 19$	$31 \pm 2 \pm 4$	$534 \pm 3 \pm 20$	543
$4\mathrm {e}$	$205 \pm 1 \pm 12$	$20 \pm 2 \pm 2$	$225 \pm 2 \pm 13$	220
Total	$1009 \pm 3 \pm 36$	$60 \pm 3 \pm 8$	$1070 \pm 4 \pm 37$	1098

The observed yields are used to evaluate the $\mathrm {p}\mathrm {p} \rightarrow \mathrm{Z}\rightarrow 4\ell$ and $\mathrm {p}\mathrm {p} \rightarrow \mathrm{Z}\mathrm{Z} \rightarrow 4\ell$ production cross sections from a combined fit to the number of observed events in all the final states. The likelihood is a combination of individual channel likelihoods for the signal and background hypotheses with the statistical and systematic uncertainties in the form of scaling nuisance parameters. The fiducial cross section is measured by scaling the cross section in the simulation by the ratio of the measured and predicted event yields given by the fit.

The definitions for the fiducial phase spaces for the $\mathrm{Z}\rightarrow 4\ell$ and $\mathrm{Z}\mathrm{Z} \rightarrow 4\ell$ cross section measurements are given in Table 4. In the $\mathrm{Z}\mathrm{Z} \rightarrow 4\ell$ case, the $\mathrm{Z}$ bosons used in the fiducial definition are built by pairing final-state leptons using the same algorithm as is used to build $\mathrm{Z}$ boson candidates from reconstructed leptons. The generator-level leptons used for the fiducial cross section calculation are “dressed” by adding the momenta of generator-level photons within $\Delta R\left( \ell ,\gamma \right) < 0.1$ to their momenta.

**Table 4:** Fiducial definitions for the reported cross sections. The common requirements are applied for both measurements

Cross section measurement	Fiducial requirements
Common requirements	$p_{\mathrm {T}} ^{\ell _1} > 20\,\text {GeV}$, $p_{\mathrm {T}} ^{\ell _2} > 10\,\text {GeV}$, $p_{\mathrm {T}} ^{\ell _{3,4}} > 5\,\text {GeV}$,
	$|\eta ^{\ell } | < 2.5$, $m_{\ell \ell } > 4\,\text {GeV}$ (any opposite-sign same-flavor pair)
$\mathrm{Z}\rightarrow 4\ell$	$m_{\mathrm{Z}_1} > 40\,\text {GeV}$
	$$80< m_{4\ell } < 100\,\text {GeV} $$
$\mathrm{Z}\mathrm{Z}\rightarrow 4\ell$	$60< \left( m_{\mathrm{Z}_1}, m_{\mathrm{Z}_2}\right) < 120\,\text {GeV}$

The measured cross sections are

2 $$\begin{aligned} \begin{aligned} \sigma _{\text {fid}}&(\mathrm {p}\mathrm {p} \rightarrow \mathrm{Z}\rightarrow 4\ell ) \\&= 31.2 _{-1.4}^{+1.5} \,\text {(stat)} _{-1.9}^{+2.1} \,\text {(syst)} \pm 0.8 \,\text {(lumi)} \text { fb},\\ \sigma _{\text {fid}}&(\mathrm {p}\mathrm {p} \rightarrow \mathrm{Z}\mathrm{Z} \rightarrow 4\ell ) \\&= 40.9 \pm 1.3 \,\text {(stat)} \pm 1.4 \,\text {(syst)} \pm 1.0 \,\text {(lumi)} \text { fb}. \end{aligned} \end{aligned}$$

The $\mathrm {p}\mathrm {p} \rightarrow \mathrm{Z}\rightarrow 4\ell$ fiducial cross section can be compared to $27.9^{+1.0}_{-1.5} \pm 0.6\text { fb}$ calculated at NLO in QCD with powheg using the same settings as used for the simulated sample described in Section 3, with dynamic scales $\mu _\mathrm {F} = \mu _\mathrm {R} = m_{4\ell }$. The uncertainties correspond to scale and PDF variations, respectively. The $\mathrm{Z}\mathrm{Z}$ fiducial cross section can be compared to $34.4^{+0.7}_{-0.6} \pm 0.5\text { fb}$ calculated with powheg and mcfm using the same settings as the simulated samples, or to $36.0_{-0.8}^{+0.9}$ computed with matrix at NNLO. The powheg and matrix calculations used dynamic scales $\mu _\mathrm {F} = \mu _\mathrm {R} = m_{4\ell }$, while the contribution from mcfm was computed with dynamic scales $\mu _\mathrm {F} = \mu _\mathrm {R} = 0.5 m_{4\ell }$.

The $\mathrm {p}\mathrm {p} \rightarrow \mathrm{Z}\rightarrow 4\ell$ fiducial cross section is scaled to $\sigma (\mathrm {p}\mathrm {p} \rightarrow \mathrm{Z}) \mathcal {B} (\mathrm{Z}\rightarrow 4\ell )$ using the acceptance correction factor $\mathcal {A} = 0.125 \pm 0.002$, estimated with powheg. This factor corrects the fiducial $\mathrm{Z}\rightarrow 4\ell$ cross section to the phase space with only the 80–100$\,\text {GeV}$ mass window and $m_{\ell ^+\ell ^-} > 4\,\text {GeV}$ requirements, and also includes a correction, $0.96 \pm 0.01$, for the contribution of nonresonant four-lepton production to the signal region. The uncertainty takes into account the interference between doubly- and singly-resonant diagrams. The measured cross section is

3 $$\begin{aligned} \sigma (\mathrm {p}\mathrm {p} \rightarrow \mathrm{Z}) \mathcal {B}(\mathrm{Z}\rightarrow 4\ell ) = 249 \pm 11 (stat) _{-15}^{+16} (syst) \pm 4 (theo) \pm 6 (lumi) fb \end{aligned}$$

The branching fraction for the $\mathrm{Z}\rightarrow 4\ell$ decay, $\mathcal {B}(\mathrm{Z}\rightarrow 4\ell )$, is measured by comparing the cross section given by Eq. (3) with the $\mathrm{Z}\rightarrow \ell ^+\ell ^-$ cross section, and is computed as

4 $$\begin{aligned} \mathcal {B}(\mathrm{Z}\rightarrow 4\ell ) = \frac{\sigma (\mathrm {p}\mathrm {p} \rightarrow \mathrm{Z}\rightarrow 4\ell )}{\mathcal {C}^{\text {60--120}}_{\text {80--100}} \, \sigma (\mathrm {p}\mathrm {p} \rightarrow \mathrm{Z}\rightarrow \ell ^+\ell ^-) / \mathcal {B}(\mathrm{Z}\rightarrow \ell ^+\ell ^-)}, \end{aligned}$$

where $\sigma (\mathrm {p}\mathrm {p} \rightarrow \mathrm{Z}\rightarrow \ell ^+\ell ^-) = 1870 _{-40}^{+50}\text { pb}$ is the $\mathrm{Z}\rightarrow \ell ^+\ell ^-$ cross section times branching fraction calculated at NNLO with fewz v2.0 [50] in the mass range 60–120$\,\text {GeV}$. Its uncertainty includes PDF uncertainties and uncertainties in $\alpha _s$, the charm and bottom quark masses, and the effect of neglected higher-order corrections to the calculation. The factor $\mathcal {C}^{\text {60--120}}_{\text {80--100}} = 0.926 \pm 0.001$ corrects for the difference in $\mathrm{Z}$ boson mass windows and is estimated using powheg. Its uncertainty includes scale and PDF variations. The nominal $\mathrm{Z}$ to dilepton branching fraction $\mathcal {B}(\mathrm{Z}\rightarrow \ell ^+\ell ^-)$ is 0.03366 [46]. The measured value is

5 $$\begin{aligned} \mathcal {B}(\mathrm{Z}\rightarrow 4\ell ) = 4.83 _{-0.22}^{+0.23} (stat) _{-0.29}^{+0.32} (syst) \pm 0.08 (theo) \pm 0.12 (lumi) \times 10^{-6} \end{aligned}$$

where the theoretical uncertainty includes the uncertainties in $\sigma (\mathrm {p}\mathrm {p} \rightarrow \mathrm{Z}) \mathcal {B} (\mathrm{Z}\rightarrow \ell ^+\ell ^-)$, $\mathcal {C}^{\text {60--120}}_{\text {80--100}}$, and $\mathcal {A}$. This can be compared with $4.6 \times 10^{-6}$, computed with MadGraph 5_amc@nlo, and is consistent with the CMS and ATLAS measurements at $\sqrt{s} = 7, 8,$ and 13$\,\text {TeV}$  [6, 14, 15].

The total $\mathrm{Z}\mathrm{Z}$ production cross section for both dileptons produced in the mass range 60–120$\,\text {GeV}$ and $m_{\ell ^+\ell ^{\prime -}} > 4\,\text {GeV}$ is found to be

6 $$\begin{aligned} \sigma (\mathrm {p}\mathrm {p} \rightarrow \mathrm{Z}\mathrm{Z}) = 17.5 _{-0.5}^{+0.6} \,\text {(stat)} \pm 0.6 \,\text {(syst)} \pm 0.4 \,\text {(theo)} \pm 0.4 \,\text {(lumi)} \text { pb}. \end{aligned}$$

The measured total cross section can be compared to the theoretical value of $14.5^{+0.5}_{-0.4} \pm 0.2\text { pb}$ calculated with a combination of powheg and mcfm with the same settings as described for $\sigma _{\text {fid}} (\mathrm {p}\mathrm {p} \rightarrow \mathrm{Z}\mathrm{Z} \rightarrow 4\ell )$. It can also be compared to $16.2^{+0.6}_{-0.4}$ $\text { pb}$, calculated at NNLO in QCD via matrix v1.0.0_beta4 [11, 51], or $15.0^{+0.7}_{-0.6} \pm 0.2$ $\text { pb}$, calculated with mcfm at NLO in QCD with additional contributions from LO $\mathrm{g} \mathrm{g} \rightarrow \mathrm{Z}\mathrm{Z}$ diagrams. Both values are calculated with the NNPDF3.0 PDF sets, at NNLO and NLO, respectively, and fixed scales set to $\mu _\mathrm {F} = \mu _\mathrm {R} = m_\mathrm{Z}$.

This measurement agrees with the previously published cross section measured by CMS at 13$\,\text {TeV}$  [6] based on a 2.6$\,\text {fb}^{-1}$ data sample collected in 2015:

7 $$\begin{aligned} \sigma (\mathrm {p}\mathrm {p} \rightarrow \mathrm{Z}\mathrm{Z}) = 14.6 ^{+1.9}_{-1.8} \,\text {(stat)} _{-0.5}^{+0.3} \,\text {(syst)} \pm 0.2 \,\text {(theo)} \pm 0.4 \,\text {(lumi)} \text { pb}. \end{aligned}$$

The two measurements can be combined to yield the “2015 + 2016 cross section”

8 $$\begin{aligned} \sigma (\mathrm {p}\mathrm {p} \rightarrow \mathrm{Z}\mathrm{Z}) = 17.2 \pm 0.5 \,\text {(stat)} \pm 0.7 \,\text {(syst)} \pm 0.4 \,\text {(theo)} \pm 0.4 \,\text {(lumi)} \text { pb}. \end{aligned}$$

The combination was performed once considering the experimental uncertainties to be fully correlated between the 2015 and 2016 data sets, and once considering them to be fully uncorrelated. The results were averaged, and the difference was added linearly to the systematic uncertainty in the combined cross section.

**Fig. 5:**
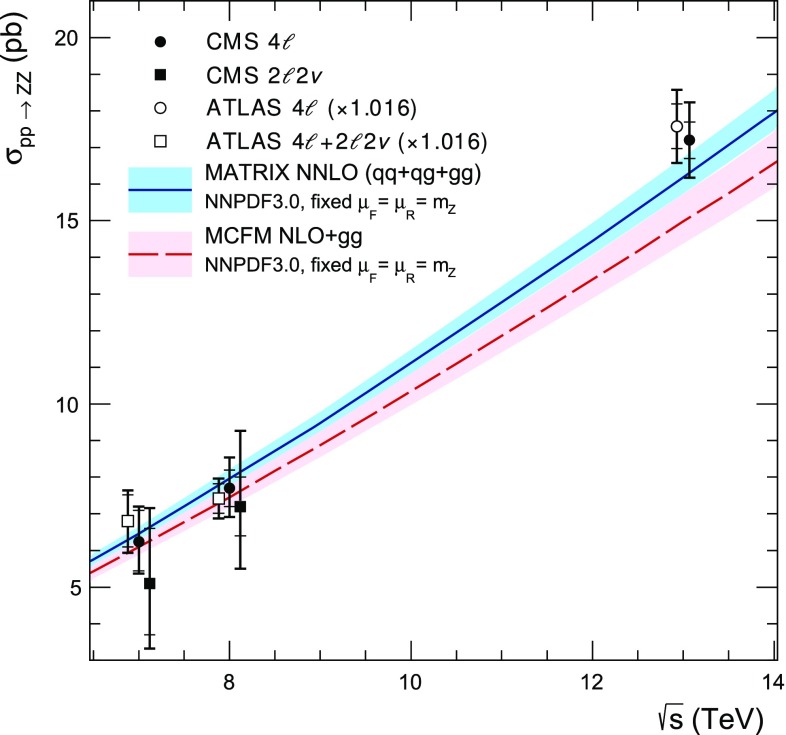
The total ZZ cross section as a function of the proton-proton center-of-mass energy. Results from the CMS and ATLAS experiments are compared to predictions from matrix at NNLO in QCD, and mcfm at NLO in QCD. The mcfm prediction also includes gluon-gluon initiated production at LO in QCD. Both predictions use NNPDF3.0 PDF sets and fixed scales $\mu _\mathrm {F} = \mu _\mathrm {R} = m_\mathrm{Z}$. Details of the calculations and uncertainties are given in the text. The ATLAS measurements were performed with a $\mathrm{Z}$ boson mass window of 66–116$\,\text {GeV}$, and are corrected for the resulting 1.6% difference. Measurements at the same center-of-mass energy are shifted slightly along the horizontal axis for clarity

The total $\mathrm{Z}\mathrm{Z}$ cross section is shown in Fig. 5 as a function of the proton-proton center-of-mass energy. Results from CMS [3, 4] and ATLAS [7, 8, 10] are compared to predictions from matrix and mcfm with the NNPDF3.0 PDF sets and fixed scales $\mu _\mathrm {F} = \mu _\mathrm {R} = m_\mathrm{Z}$. The matrix prediction uses PDFs calculated at NNLO, while the mcfm prediction uses NLO PDFs. The uncertainties are statistical (inner bars) and statistical and systematic added in quadrature (outer bars). The band around the matrix predictions reflects scale uncertainties, while the band around the mcfm predictions reflects both scale and PDF uncertainties.

The measurement of the differential cross sections provides detailed information about $\mathrm{Z}\mathrm{Z}$ kinematics. The observed yields are unfolded using the iterative technique described in Ref. [52]. Unfolding is performed with the RooUnfold package [53] and regularized by stopping after four iterations. Statistical uncertainties in the data distributions are propagated through the unfolding process to give the statistical uncertainties on the normalized differential cross sections.

The three decay channels, $4\mathrm {e}$, $4\mathrm {\mu }$, and $2\mathrm {e}2\mathrm {\mu }$, are combined after unfolding because no differences are expected in their kinematic distributions. The generator-level leptons used for the unfolding are dressed as in the fiducial cross section calculation.

**Fig. 6:**
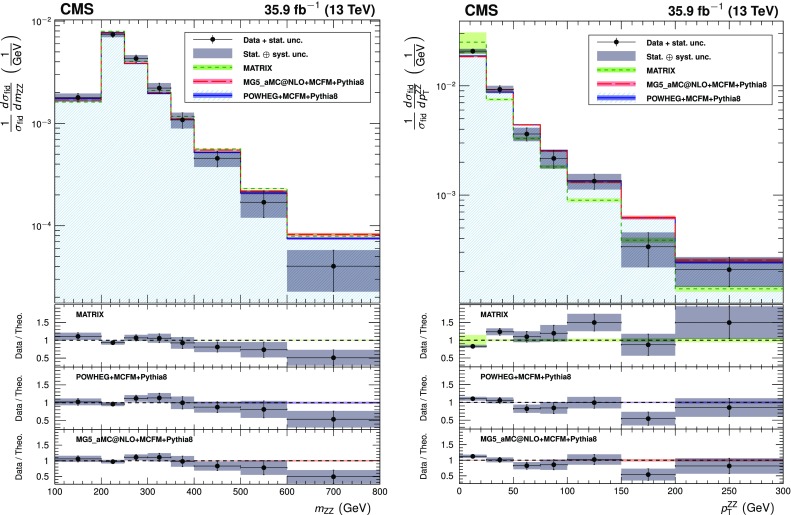
Differential cross sections normalized to the fiducial cross section for the combined 4$\mathrm {e}$, 4$\mathrm {\mu }$, and 2$\mathrm {e}$2$\mathrm {\mu }$ decay channels as a function of mass (left) and $p_{\mathrm {T}}$ (right) of the $\mathrm{Z}\mathrm{Z}$ system. Points represent the unfolded data; the solid, dashed, and dotted histograms represent the powheg+mcfm, MadGraph 5_amc@nlo+mcfm, and matrix predictions for $\mathrm{Z} \mathrm{Z}$ signal, respectively, and the bands around the predictions reflect their combined statistical, scale, and PDF uncertainties pythia  v8 was used for parton showering, hadronization, and underlying event simulation in the powheg, MadGraph 5_amc@nlo, and mcfm samples. The lower part of each plot represents the ratio of the measured cross section to the theoretical distributions. The shaded grey areas around the points represent the sum in quadrature of the statistical and systematic uncertainties, while the crosses represent the statistical uncertainties only

**Fig. 7:**
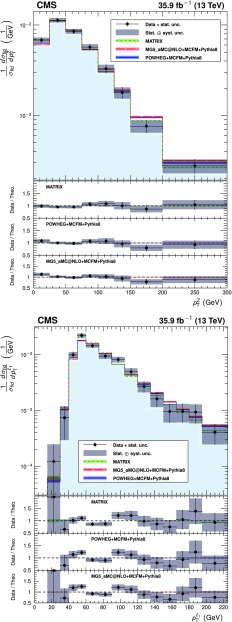
Normalized $\mathrm{Z}\mathrm{Z}$ differential cross sections as a function of the $p_{\mathrm {T}}$ of (upper) all $\mathrm{Z}$ bosons and (lower) the leading lepton in $\mathrm{Z}\mathrm{Z}$ events. Other details are as described in the caption of Fig. 6

**Fig. 8:**
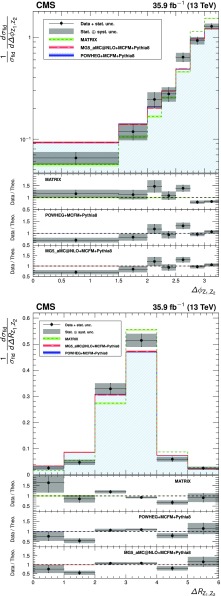
Normalized $\mathrm{Z}\mathrm{Z}$ differential cross sections as a function of (upper) the azimuthal separation of the two $\mathrm{Z}$ bosons and (lower) $\Delta R$ between the $\mathrm{Z}$-bosons. Other details are as described in the caption of Fig. 6

The differential distributions normalized to the fiducial cross sections are presented in Figs. 6, 7, 8 for the combination of the 4$\mathrm {e}$, 4$\mathrm {\mu }$, and 2$\mathrm {e}$2$\mathrm {\mu }$ decay channels. The fiducial cross section definition includes $p_{\mathrm {T}} ^{\ell }$ and $|\eta ^{\ell } |$ selections on each lepton, and the 60–120$\,\text {GeV}$ mass requirement, as described in Table 4 and Sect. 4. Figure 6 shows the normalized differential cross sections as functions of the mass and $p_{\mathrm {T}}$ of the $\mathrm{Z}\mathrm{Z}$ system, Fig. 7 shows them as functions of the $p_{\mathrm {T}}$ of all $\mathrm{Z}$ bosons and the $p_{\mathrm {T}}$ of the leading lepton in each event, and Fig. 8 shows the angular correlations between the two $\mathrm{Z}$ bosons. The data are corrected for background contributions and compared with the theoretical predictions from powheg and mcfm, MadGraph 5_amc@nlo and mcfm, and matrix. The bottom part of each plot shows the ratio of the measured to the predicted values. The bin sizes are chosen according to the resolution of the relevant variables, while also keeping the statistical uncertainties at a similar level in all bins. The data are well reproduced by the simulation except in the low $p_{\mathrm {T}}$ regions, where data tend to have a steeper slope than the prediction.

**Fig. 9:**
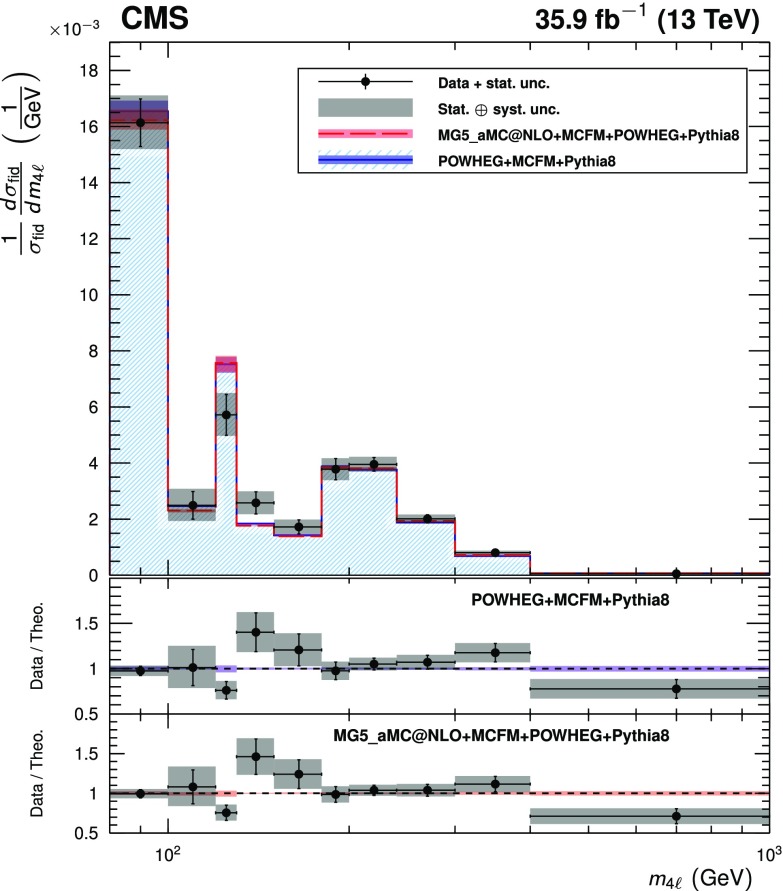
The normalized differential four-lepton cross section as a function of the four-lepton mass, subject only to the common requirements of Table 4. SM $\mathrm{g} \mathrm{g} \rightarrow \mathrm{H} \rightarrow \mathrm{Z}\mathrm{Z}^*$ production is included, simulated with powheg. Other details are as described in the caption of Fig. 6

Figure 9 shows the normalized differential four-lepton cross section as a function of $m_{4\ell }$, subject only to the common requirements of Table 4. This includes contributions from the $\mathrm{Z}$ and Higgs boson resonances and continuum $\mathrm{Z}$ $\mathrm{Z}$ production.

## Limits on anomalous triple gauge couplings

**Fig. 10:**
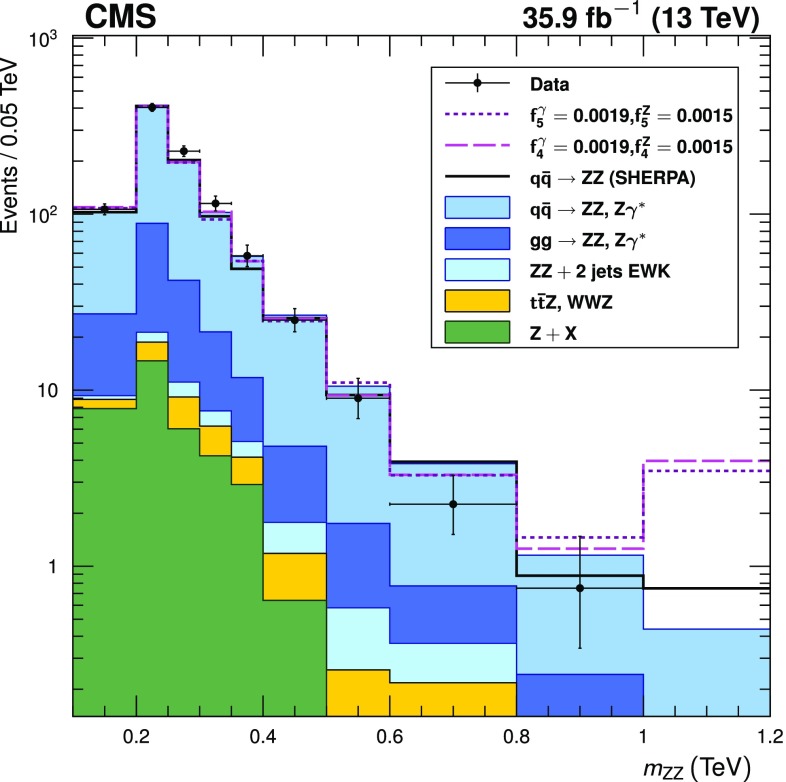
Distribution of the four-lepton reconstructed mass for the combined $4\mathrm {e}$, 4$\mathrm {\mu }$, and $2\mathrm {e}2\mathrm {\mu }$ channels. Points represent the data, the filled histograms represent the SM expected yield including signal and irreducible background predictions from simulation and the data-driven background estimate. Unfilled histograms represent examples of aTGC signal predictions (dashed), and the sherpa SM prediction (solid), included to illustrate the expected shape differences between the sherpa and powheg predictions. Vertical bars on the data points show their statistical uncertainty. The sherpa distributions are normalized such that the SM sample has the same total yield as the powheg sample predicts. Bin contents are normalized to the bin widths, using a unit bin size of 50$\,\text {GeV}$; horizontal bars on the data points show the range of the corresponding bin. The last bin includes the “overflow” contribution from events at masses above 1.2$\,\text {TeV}$

The presence of aTGCs would increase the yield of events at high four-lepton masses. Figure 10 presents the distribution of the four-lepton reconstructed mass of events with both $\mathrm{Z}$ bosons in the mass range 60–120$\,\text {GeV}$ for the combined $4\mathrm {e}$, 4$\mathrm {\mu }$, and $2\mathrm {e}2\mathrm {\mu }$ channels. This distribution is used to set the limits on possible contributions from aTGCs. Two simulated samples with nonzero aTGCs are shown as examples, along with the SM distribution simulated by both sherpa and powheg.

**Fig. 11:**
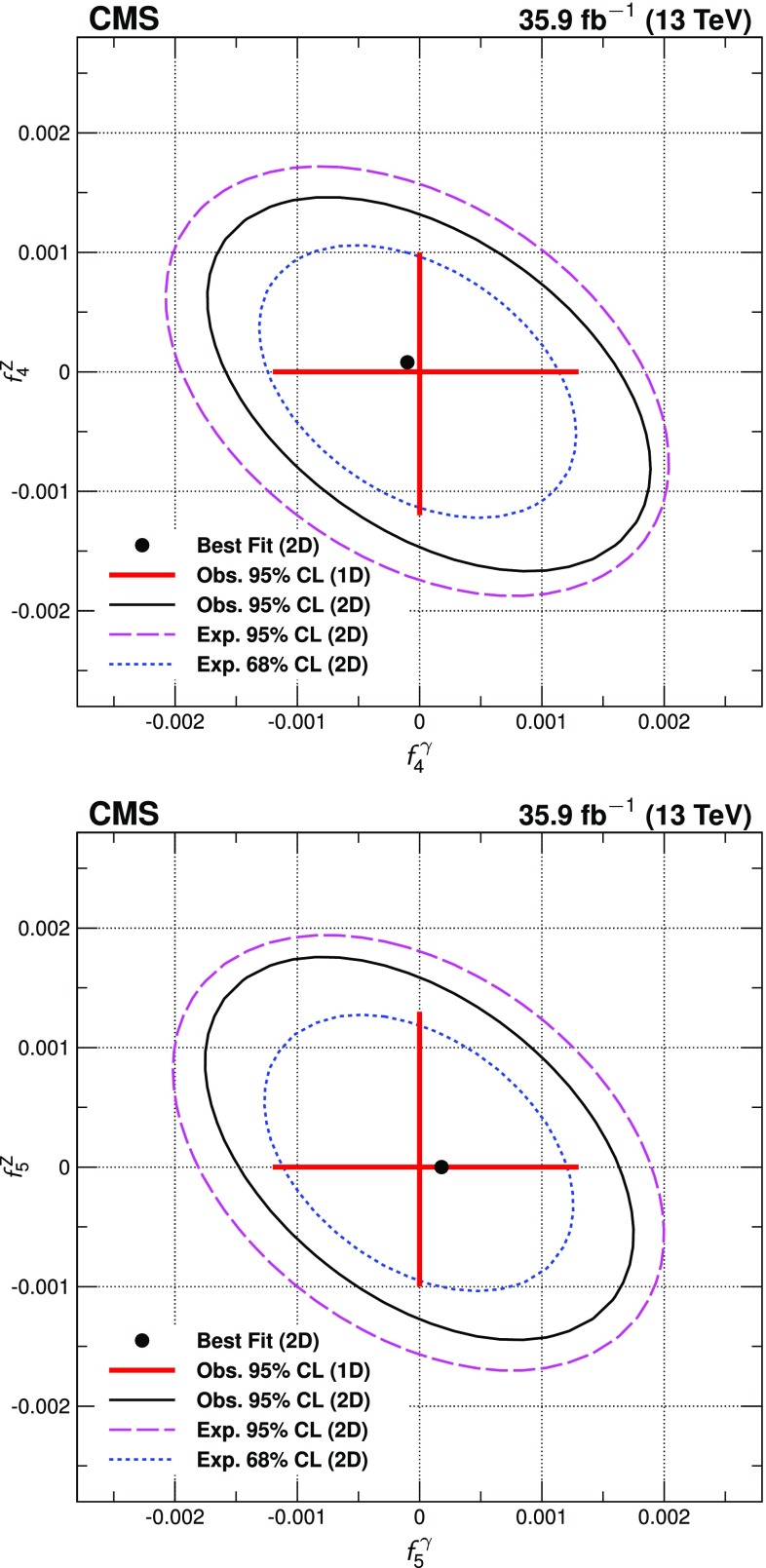
Two-dimensional observed 95% CL limits (solid contour) and expected 68 and 95% CL limits (dashed contour) on the $\mathrm{Z}\mathrm{Z}\mathrm{Z}$ and $\mathrm{Z}\mathrm{Z}\gamma$ aTGCs. The upper(lower) plot shows the exclusion contour in the $f_{4(5)}^\mathrm{Z}, f_{4(5)}^\gamma$ parameter planes. The values of couplings outside of contours are excluded at the corresponding confidence level. The solid dot is the point at which the likelihood is at its maximum. The solid lines at the center show the observed one-dimensional 95% CL limits for $f_{4,5}^\gamma$ (horizontal) and $f_{4,5}^\mathrm{Z}$ (vertical). No form factor is used

The invariant mass distributions are interpolated from the sherpa simulations for different values of the anomalous couplings in the range between 0 and 0.015. For each distribution, only one or two couplings are varied while all others are set to zero. The measured signal is obtained from a comparison of the data to a grid of aTGC models in the $(f_{4}^\mathrm{Z}, f_{4}^\gamma )$ and $(f_{5}^\mathrm{Z}, f_{5}^\gamma )$ parameter planes. Expected signal values are interpolated between the 2D grid points using a second-degree polynomial, since the cross section for the signal depends quadratically on the coupling parameters. A binned profile likelihood method, Wald Gaussian approximation, and Wilk’s theorem are used to derive one-dimensional limits at a 95% confidence level (CL) on each of the four aTGC parameters, and two-dimensional limits at a 95% CL on the pairs ($f_4^\mathrm{Z}$, $f_4^\gamma$) and ($f_5^\mathrm{Z}$, $f_5^\gamma$) [46, 54, 55]. When the limits are calculated for each parameter or pair, all other parameters are set to their SM values. The systematic uncertainties described in Section 7 are treated as nuisance parameters with log-normal distributions. No form factor is used when deriving the limits so that the results do not depend on any assumed energy scale characterizing new physics. The constraints on anomalous couplings are displayed in Fig. 11. The curves indicate 68 and 95% confidence levels, and the solid dot shows the coordinates where the likelihood reaches its maximum. Coupling values outside the contours are excluded at the corresponding confidence levels. The limits are dominated by statistical uncertainties.

The observed one-dimensional 95% CL limits for the $f_4^{\mathrm{Z},\gamma }$ and $f_5^{\mathrm{Z},\gamma }$ anomalous coupling parameters are:

9 $$\begin{aligned} \begin{aligned} -0.0012<f_4^\mathrm{Z}< 0.0010 ,\quad -0.0010<f_5^\mathrm{Z}< 0.0013 , \\ \quad -0.0012<f_4^{\gamma }< 0.0013 ,\quad -0.0012<f_5^{\gamma }< 0.0013 . \end{aligned} \end{aligned}$$

These are the most stringent limits to date on anomalous $\mathrm{Z}\mathrm{Z}\mathrm{Z}$ and $\mathrm{Z}\mathrm{Z}\gamma$ trilinear gauge boson couplings, improving on the previous strictest results from CMS [5] by factors of two or more and constraining the coupling parameters more than the corresponding ATLAS results [10].

**Fig. 12:**
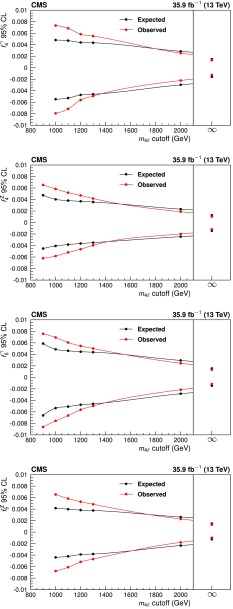
Expected and observed one-dimensional limits on the four aTGC parameters, as a function of an upper cutoff on the invariant mass of the four-lepton system. No form factor is used

One way to impose unitarity on the aTGC models is to restrict the range of four-lepton invariant mass used in the limit calculation. The limits will then depend on the “cutoff” value used. The computation of the one-dimensional limits is repeated for different maximum allowed values of $m_{4\ell }$, and the results are presented in Fig. 12 as a function of this cutoff.

## Summary

A series of measurements of four-lepton final states in proton-proton collisions at $\sqrt{s} = 13\,\text {TeV}$ have been performed with the CMS detector at the CERN LHC. The measured $\mathrm {p}\mathrm {p} \rightarrow \mathrm{Z}\mathrm{Z}$ cross section is $\sigma (\mathrm {p}\mathrm {p}\rightarrow \mathrm{Z}\mathrm{Z}) = 17.2 \pm 0.5 \,\text {(stat)} \pm 0.7 \,\text {(syst)} \pm 0.4 \,\text {(theo)} \pm 0.4 \,\text {(lumi)} \text { pb}$ for $\mathrm{Z}$ boson masses in the range $60< m_{\mathrm{Z}} < 120\,\text {GeV}$. The measured branching fraction for $\mathrm{Z}$ boson decays to four leptons is $\mathcal {B}(\mathrm{Z}\rightarrow 4\ell ) = 4.83 _{-0.22}^{+0.23} (stat)_{-0.29}^{+0.32} (syst) \pm 0.08 (theo) \pm 0.12 (lumi) \times 10^{-6}$ for four-lepton mass in the range $80< m_{4\ell } < 100\,\text {GeV}$ and dilepton mass $m_{\ell \ell } > 4\,\text {GeV}$ for all oppositely charged same-flavor lepton pairs. Normalized differential cross sections were also measured. All results agree well with the SM predictions. Improved limits on anomalous $\mathrm{Z} \mathrm{Z} \mathrm{Z}$ and $\mathrm{Z} \mathrm{Z} \gamma$ triple gauge couplings were established, the most stringent to date.
